# Role of the prefrontal cortical protease TACE/ADAM17 in neurobehavioral responses to chronic stress during adolescence

**DOI:** 10.1002/brb3.3482

**Published:** 2024-05-07

**Authors:** Fransua Sharafeddin, Julio Sierra, Mina Ghaly, Timothy B. Simon, Perla Ontiveros‐Ángel, Brandon Edelbach, Marcelo Febo, Jennifer Labus, Johnny D. Figueroa

**Affiliations:** ^1^ Center for Health Disparities and Molecular Medicine Loma Linda University School of Medicine Loma Linda California USA; ^2^ Department of Basic Sciences Loma Linda University School of Medicine Loma Linda California USA; ^3^ Translational Research Imaging Laboratory, Department of Psychiatry, Department of Neuroscience, College of Medicine University of Florida Health Gainesville Florida USA; ^4^ Graduate Program in Bioscience, Division of Digestive Diseases, David Geffen School of Medicine University of California Los Angeles USA; ^5^ Department of Neurosurgery Loma Linda University School of Medicine Loma Linda CA USA

**Keywords:** chronic adolescent stress, home‐cage monitoring, naturalistic behaviors, prefrontal cortex, RNA silencing

## Abstract

**Introduction:**

Chronic adolescent stress profoundly affects prefrontal cortical networks regulating top‐down behavior control. However, the neurobiological pathways contributing to stress‐induced alterations in the brain and behavior remain largely unknown. Chronic stress influences brain growth factors and immune responses, which may, in turn, disrupt the maturation and function of prefrontal cortical networks. The tumor necrosis factor alpha‐converting enzyme/a disintegrin and metalloproteinase 17 (TACE/ADAM17) is a sheddase with essential functions in brain maturation, behavior, and inflammatory responses. This study aimed to determine the impact of stress on the prefrontal cortex and whether TACE/ADAM17 plays a role in these responses.

**Methods:**

We used a Lewis rat model that incorporates critical elements of chronic psychosocial stress, such as uncontrollability, unpredictability, lack of social support, and re‐experiencing of trauma.

**Results:**

Chronic stress during adolescence reduced the acoustic startle reflex and social interactions while increasing extracellular free water content and TACE/ADAM17 mRNA levels in the medial prefrontal cortex. Chronic stress altered various ethological behavioral domains in the observation home cages (decreased ingestive behaviors and increased walking, grooming, and rearing behaviors). A group of rats was injected intracerebrally either with a novel Accell™ SMARTpool TACE/ADAM17 siRNA or a corresponding siRNA vehicle (control). The RNAscope Multiplex Fluorescent v2 Assay was used to visualize mRNA expression. Automated puncta quantification and analyses demonstrated that TACE/ADAM17 siRNA administration reduced TACE/ADAM17 mRNA levels in the medial prefrontal cortex (59% reduction relative to control). We found that the rats that received prefrontal cortical TACE/ADAM17 siRNA administration exhibited altered eating patterns (e.g., increased food intake and time in the feeding zone during the light cycle).

**Conclusion:**

This study supports that the prefrontal cortex is sensitive to adolescent chronic stress and suggests that TACE/ADAM17 may be involved in the brain responses to stress.

## Graphical abstract

This study delved into the repercussions of chronic psychosocial stress during adolescence on prefrontal cortical networks, revealing shifts in behavior, brain, and molecular markers. Utilizing a Lewis rat model, the research reports a reduction in acoustic startle reflex and social interactions, coupled with elevated extracellular free water content and TACE/ADAM17 mRNA levels in the medial prefrontal cortex under chronic psychosocial stress conditions. Employing automated observational cages alongside statistical data transformation facilitated a deeper understanding of stress‐induced alterations, and discovered new roles for prefrontal cortical TACE/ADAM17.

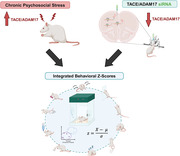

## 1 INTRODUCTION

Chronic stress is a pervasive concern affecting individuals of all ages, but its profound implications for the developing adolescent brain are increasingly recognized as a critical area of study (Carbone et al., 2019; Cisler & Herringa, 2021; McKnight‐Eily et al., 2021). Accumulating evidence from human and animal studies highlights the multifaceted impact of prolonged stress exposure on the developing brain (Cisler & Herringa, 2021; Page & Coutellier, 2018; Zhu & Grace, 2023). Yet, there is an urgent need to understand the molecular players influencing brain structure and behavior in youth exposed to chronic stress. Understanding the intricate relationship between adolescent chronic stress and its consequences on both brain structure and behavior is paramount for elucidating the long‐term mental health outcomes in this vulnerable population.

The prefrontal cortex (PFC) is a cortical region with some of the most substantial remodeling during adolescence (Sowell et al., 2003). The PFC is central in regulating cognition and behavior (Catani, 2019). This brain region is associated with the higher‐order cognitive and social‐emotional functions and is responsible for conducting complex goal‐directed activities representing the executive function (Henri‐Bhargava et al., 2018). In particular, the medial prefrontal cortex (mPFC) is implicated in cognitive function, social and feeding behaviors, food valuation, motivation, and emotional regulation (Xu et al., 2019). It is noteworthy that the mPFC, characterized by its prolonged maturation timeline compared to other cortical regions, exhibits heightened susceptibility to the effects of adolescent stress. Nevertheless, there remains a significant gap in our understanding regarding the precise molecular mechanisms through which chronic adolescent stress disrupts the maturation and function of the mPFC.

The development of synaptic networks extends into the postnatal period, encompassing a multifaceted process involving synaptogenesis and synaptic pruning that occurs simultaneously, exerting a profound influence on the structural organization of mPFC circuits (Collin & Heuvel, 2013). This intricate synaptic refinement mechanism is facilitated by microglia, the brain's innate immune cells, playing a pivotal role in sculpting the maturing PFC (Mallya et al., 2019; Schalbetter et al., 2022). The orchestration of this process relies on intricate regulatory pathways that encompass both permissive and repulsive growth signals, governing the molecular signaling cascades underpinning synaptic maturation (Arx et al., 2023; Jay et al., 2019; Tagliatti et al., 2024). Central to these mechanisms is the tumor necrosis factor alpha‐converting enzyme/a disintegrin and metalloproteinase 17 (TACE/ADAM17), a key sheddase responsible for cleaving various growth factors and inflammatory mediators (Zhang et al., 2016). TACE/ADAM17 has been implicated in a spectrum of diseases, spanning heart failure, diabetes, cancer, atherosclerosis, arthritis, and central nervous system pathologies (Chemaly et al., 2017; Zhang et al., 2016). Given its pivotal role in maintaining microglial survival and phagocytic functions (Sommer et al., 2019; Vidal et al., 2013), we have recently put forth a model positing that excessive TACE/ADAM17 activities may disrupt normal brain maturation (Vega‐Torres et al., 2022). Here, we reasoned that TACE/ADAM17 might contribute to behavioral alterations associated with adolescent chronic stress (Rose‐John, 2013; Yoon & Baik, 2013; Zunke & Rose‐John, 2017). By examining the effects of chronic stress on adolescent brain structure and behavior, this study aimed to elucidate the nuanced pathways underlying these alterations, ultimately contributing to a deeper understanding of the long‐term implications of stress on mental and emotional well‐being. Our novel approaches and findings serve as a foundation for further investigation into chronic stress mitigation and intervention strategies targeted at this critical stage of life.

## 2 MATERIALS AND METHODS

### 2.1 Rat model

All experimental procedures adhered to animal protocol 20–171, which received approval from the Institutional Animal Care and Use Committee (IACUC) at the Loma Linda University School of Medicine. The animals were maintained in standard housing conditions, with a temperature of 21 ± 2°C, relative humidity set at 45%, and a light‐dark cycle of 12 h, with lights on at 7:00 AM. Control groups were pair‐housed for the duration of the study. The body weights of the animals were routinely recorded on a weekly or daily basis, specifically during the week of behavioral testing. Food consumption was quantified at least twice a week, and it is crucial to note that the rats were never subjected to food or water restriction. Lewis rats were employed for this study, as they have previously demonstrated reduced activation of the hypothalamic‐pituitary‐adrenocortical (HPA) axis in response to stress. These characteristics mimic neurophysiological processes in humans exposed to trauma, making this rat model suitable for our proposed experiments (Carpenter et al., 2007; Miller et al., 2007). We and others have used Lewis rats to model abnormal neuronal maturation in adolescence due to obesogenic diet exposure (Vega‐Torres et al., 2018); genetic influence on addiction vulnerability, with particular emphasis on differences in mesolimbic dopamine transmission, rewarding and emotional function (Cadoni, 2016); high‐fat diet‐induced increase in susceptibility to traumatic stress during adolescence (Kalyan‐Masih et al., 2016).

### 2.2 Stress model

The psychosocial stress (PSS) protocol was adapted from an established rat model of traumatic stress. PSS produces fear and anxiety‐like behaviors lasting up to 4 months (Zoladz et al., 2012; Zoladz et al., 2015). PSS consisted of subjecting immobilized rats to two 1‐h‐long exposures to a domestic cat. To achieve immobilization, the rats were placed in plastic DecapiCones (Cat. No. NC9679094, Braintree Scientific; MA, USA) and positioned inside a perforated wedge‐shaped plexiglass pie cage designed for aerosol delivery (Cat. No. RPC‐1 AERO, Braintree Scientific; MA, USA; dimensions: diameter 41 cm × height 6.75 cm). A container filled with soiled cat litter was connected to a nebulizer, aiding in the dispersal of aerosols. This setup was situated within a metal enclosure (dimensions: 91.4 cm × 58.4 cm × 63.5 cm, Amazon Basics, Amazon, USA) alongside the freely moving cat.

The stress exposures were administered on 2 distinct days: the first occurred during the dark cycle on day 1, and the second occurred during the light cycle on day 10 of the PSS protocol. The animals in the stress group experienced social isolation, involving solitary housing throughout both the stress perturbation and experimentation periods. This model replicates critical aspects of trauma experienced by humans, including the loss of control over stressful circumstances and the inability to predict forthcoming events. Furthermore, it encompasses elements of loneliness, social isolation, and a lack of social support, which are essential psychosocial components observed in stress‐related disorders.

### 2.3 Adolescence model

In this study, rat adolescence was defined in accordance with the parameters established by Spear (2000), indicating that the onset typically occurs around postnatal day 28 (PND28), with potential variations, notably earlier onset in females. This developmental phase can persist until PND60 or beyond, particularly in male rats. Similar to humans, delineating precise boundaries for the onset and conclusion of adolescence in rats is challenging, as the initial emergence of adolescent traits and the persistence of residual characteristics are not distinctly defined. Our study specifically targeted adolescence (PND28‐PND60) rather than the narrower timeframe associated with puberty. Previously unpublished research conducted in our laboratory revealed heightened TACE/ADAM17 mRNA levels in the medial prefrontal cortex (mPFC) of mid‐late adolescent rats compared to their younger counterparts. Motivated by this finding and aiming for a comprehensive exploration of the effects of stress on the brain, behavior, and the potential role of TACE/ADAM17, we embarked on a series of three studies. Study 1 was meticulously designed to delve into the ramifications of stress spanning from late adolescence through adulthood, probing the enduring effects of stress exposure during this critical maturational window. Study 2 aimed to determine the efficacy of a passive delivery siRNA system to reduce TACE/ADAM17 expression in adolescent rats. Study 3 was tailored to hone in on the specific impact of stress during the early to mid‐adolescent period, aiming to capture the nuances of stress responses and adaptations during this formative stage of development. In all three studies, rats were subjected to stressors during adolescence, with data collection extending into adulthood. This experimental design allowed us to assess the enduring impacts of stress exposure during critical developmental periods on subsequent brain function and behavior. Additionally, Study 3 aimed to elucidate the potential of TACE/ADAM17 silencing as a means to modulate the effects of stress on the brain and behavior.

### 2.4 Study 1: Determining the consequences of chronic stress in brain and behavior

Fifty‐six male adolescent Lewis rats (postnatal day 21, PND21) were weaned and carefully matched by body weight and acoustic startle reactivity. At PND51, the rats were further subdivided based on stress exposure. Exposed groups underwent the psychosocial stress (PSS) paradigm described above (for Study 1: predator encounters and social instability). PSS effects were assessed using standard behavioral tests (described below), neuroimaging (diffusion magnetic resonance imaging, d‐MRI), and gene expression analyses (qRT‐PCR).

#### 2.4.1 Diffusion‐MRI

A subcohort of rats was methodically selected for neuroimaging investigations, comprising distinct groups: UNEX (*n* = 6) and EXP (*n* = 7). Profound anesthesia was induced in the rats via intraperitoneal administration of Euthasol (150 mg/kg; Virbac, Fort Worth, TX). Subsequently, transcardiac perfusion was meticulously executed, commencing with a preliminary wash using a solution of 9.25% sucrose in distilled water, followed by perfusion with 4% paraformaldehyde (PFA; fixative, Cat No. 15714‐S, Electron Microscopy Sciences, Hatfield, PA, USA). The Perfusion TwoTM Automated Pressure Perfusion System (Leica Biosystems, Buffalo Grove, IL) was employed for this perfusion procedure. Post‐perfusion, the brains were securely housed within the cranial cavity, subjected to further fixation in 4% PFA, thoroughly washed, and subsequently stored at a temperature of 4°C in 0.1 M PBS containing 0.05% Sodium azide until the initiation of neuroimaging. During MRI data acquisition and subsequent analysis, a double‐blind protocol was strictly adhered to, ensuring the concealment of group designations. Neuroimaging procedures are fully detailed in the Supplemental Methods and Materials section.

#### 2.4.2 qRT‐PCR

Seven rats were randomly selected from each group and humanely euthanized using Euthasol (Virbac, Fort Worth, TX, USA) and transcardial perfusion with phosphate‐buffered saline (PBS). Following the perfusion procedure, the prefrontal cortex was carefully isolated. Prefrontal cortical tissue was shipped to EpigenDx™ (Hopkinton, MA, USA) for qRT‐PCR. Total RNA extraction was conducted using 1 mL of TRIzol™ Reagent (Cat. No. 15596026, ThermoFisher Scientific, Waltham, MA, USA), following the manufacturer's recommended protocols. Subsequently, the High‐Capacity cDNA Reverse Transcription Kit (Cat. No. 4368814, Applied Biosystems, Waltham, MA, USA) was employed to synthesize a 20‐µL cDNA sample from 2 µg of RNA. For gene expression analysis, real‐time PCR was performed using TaqMan gene expression assays targeting the rat TACE/ADAM17 on the StepOnePlus™ Real‐Time PCR System (Applied Biosystems, Waltham, MA, USA). mRNA levels were determined using the ΔΔ cycle threshold method, and gene expression data were subsequently normalized to GAPDH levels.

### 2.5 Study 2: Determining prefrontal cortical TACE/ADAM17 mRNA knockdown efficiency

A total of 27 adolescent male Lewis rats, all at the postnatal day (PND) 15, were procured from Charles River Laboratories, Portage, MI. These animals were acclimated to their housing conditions, characterized by 12‐h light and dark phases, for at least 2 weeks before the onset of experimental procedures. This duration was thoughtfully selected to ensure ample time for the animals to adapt to their surroundings and the experimental conditions. Furthermore, it was aligned with the established timelines previously utilized in our laboratory, maintaining consistency in experimental procedures and data interpretation. Additionally, this timeframe accommodated the necessary surgical manipulations, allowing for proper preparation and recovery before subsequent experimental procedures. Weaning of the rats was performed at PND22. The rats were stratified into four distinct groups, predicated on the treatment administered. These groups were delineated as follows: (1) Control (*n* = 9), (2) siRNA (*n* = 10), (3) siRNA positive control (*n* = 4), (4) siRNA negative control (*n* = 4).

#### 2.5.1 Animal preparation and surgical procedure

Animals were transported to the surgical suite 30 min before the commencement of the surgical procedure. Initially, the rats were placed within an anesthesia induction chamber, and the Isoflurane flow rate was set at 4 liters per minute (lpm) to induce anesthesia. Rats were then shaved and securely positioned within the stereotactic apparatus, with ear pins firmly in place. A midline incision and a bur hole were created on the right side, corresponding to the right medial prefrontal cortex (mPFC). The stereotactic coordinates for needle insertion were 3.5 mm anterior, 0.6 mm lateral, and 4.5 mm ventral from bregma. Subsequently, the Isoflurane flow rate was adjusted to 2.5 lpm. The needle was carefully inserted into the right mPFC, and the infusion was initiated. The infusion proceeded at a rate of 0.2 µL/min over a 15‐min duration, delivering a total of 3 µL of the compound. Following completion of the infusion, the needle was left in place for an additional 10 min before being gradually withdrawn. A subcutaneous injection of 3 mL of 0.9% NaCl was administered, and the incision was closed with skin clips. The animals were then relocated to a heated recovery chamber, where they were continuously monitored for 2 h before being returned to their respective home cages.

#### 2.5.2 RNA interference

A novel Accell SMARTpool ADAM17 siRNA (Cat. No. E‐080034‐00‐0050, Horizon Discovery, Lafayette, CO, USA) was utilized, obviating the need for transfection reagents or viral vectors for delivery. This SMARTpool encompassed four distinct sequences targeting the ADAM17 gene: (1) Accell SMARTpool siRNA A‐080034‐13, Adam17, Target Sequence: GGAUUAGCUUACGUUGGUU, with a molecular weight of 13563.8 g/mol and an extinction coefficient of 356,534 L/mol·cm; (2) Accell SMARTpool siRNA A‐080034‐14, Adam17, Target Sequence: GUAUAAGUCUGAAGAUAUC, with a molecular weight of 13495.7 g/mol and an extinction coefficient of 371,664 L/mol·cm; (3) Accell SMARTpool siRNA A‐080034‐15, Adam17, Target Sequence: UCAUCGAUUUUAUAAGUAC, with a molecular weight of 13485.8 g/mol and an extinction coefficient of 375,224 L/mol·cm; and (4) Accell SMARTpool siRNA A‐080034‐16, Adam17, Target Sequence: UUAUGGAGUACAGAUAGAA, with a molecular weight of 13440.6 g/mol and an extinction coefficient of 370,062 L/mol·cm.

#### 2.5.3 Tissue collection and preparation

Following a 4‐day period after injections, the rats were anesthetized using Isoflurane and subsequently intraperitoneally injected with Euthasol (150 mg/kg; Virbac, Fort Worth, TX). After the induction of terminal anesthesia, the rats underwent transcardial perfusion utilizing the Perfusion Two™ system (Leica Biosystems, Chicago, IL). Per the manufacturer's guidelines, the prewash solution consisted of ice‐cold 9.25% sucrose solution in distilled deionized water, followed by fixation with 4% paraformaldehyde (PFA). The brains were carefully harvested and post‐fixed overnight in 4% PFA. Subsequently, the brains underwent dehydration with 30% sucrose solution in PBS at 4°C, allowing them to fully settle to the container's bottom. Following dehydration, the brains were embedded in Tissue‐Tek Optimal Cutting Temperature Compound (OCT) on dry ice and preserved at −80°C for cryosectioning.

#### 2.5.4 Cryosectioning

Before initiating the cryosectioning process, the brains were equilibrated at −20°C inside a cryostat (Leica CM3050 S, Leica Biosystems, Wetzlar, Germany). Subsequently, the brains were coronally sectioned at a thickness of 10 µm. These sections were mounted on slides and air‐dried for 60 min at −20°C to facilitate RNAscope analysis.

#### 2.5.5 RNAscope analysis

We employed the RNAscope Multiplex Fluorescent v2 Assay (Advanced Cell Diagnostics, ACD; Newark, CA) to visualize mRNA expression, enabling the simultaneous detection of up to four mRNA targets. The RNAscope target probe for Adam17 (Cat. No. 1052461‐C1) and Aif1 (Cat. No. 457731‐C3) (both from Advanced Cell Diagnostics, Inc.) was allocated to channels C1 and C3, respectively. The slides carrying brain sections were washed in Phosphate‐buffered saline (PBS) for 5 min at room temperature to eliminate OCT, followed by a 30‐min incubation at 60°C. Subsequently, the slides were post‐fixed by immersion in prechilled 4% paraformaldehyde (PFA) in PBS for 15 min at 4°C. The brain sections were then dehydrated through a series of ethanol solutions: 50% for the first immersion, 70% for the second immersion, and 100% for the third and fourth immersions, with each immersion lasting for 5 min at room temperature. After dehydration, the slides were air‐dried for 5 min at room temperature. Hydrogen peroxide was added and incubated for 10 min at room temperature. Following incubation, the slides were washed with distilled deionized water (DDW) for 30 s at room temperature. This last step was repeated with fresh DDW. Subsequently, a target retrieval was performed by immersing the slides first in boiling DDW for 10 s for acclimation and then in a boiling 1x Target Retrieval Reagent for 5 min. Next, the slides were washed in DDW for 15 s at room temperature, with subsequent immersion in a 100% ethanol solution for 3 min. The slides were then air‐dried for 5 min at room temperature. A hydrophobic barrier pen was employed to create a barrier around each brain section on the slide.

Protease III was added to each section, and the slides were incubated for 30 min at 40°C. Following incubation, the slides were washed with DDW for 2 min at room temperature, repeating the last step with fresh DDW. The Adam17 and Aif1 probes were introduced to each slide for hybridization and incubated for 2 h at 40°C. After hybridization, the slides were washed in a washing buffer for 2 min at room temperature, repeating this step with a fresh washing buffer. The slides were immersed in 5x Saline Sodium Citrate and stored overnight at 4°C.

The next day, the slides were washed in a washing buffer for 2 min at room temperature, repeating the step with fresh washing buffer, after which the amplification stage was initiated. Amp 1 solution was added to each slide and hybridized for 30 min at 40°C. Subsequently, the slides were washed in a washing buffer for 2 min at room temperature, repeating this step with fresh washing buffer. The amplification process was repeated with Amp 2 and then with Amp 3 solutions. Upon completion of the amplification stage, channel development was initiated.

The signal from channel 1 was developed by adding HRP‐C1 solution to each slide and incubating for 15 min at 40°C. The slides were then washed in a washing buffer for 2 min at room temperature, repeating the last step with fresh washing buffer. OpalTM 520 dye (FP1487001KT, Akoya Biosciences) was assigned to channel 1 and added to each slide, followed by incubation for 30 min at 40°C. Subsequently, the slides were washed in a washing buffer for 2 min at room temperature, repeating this step with fresh washing buffer. An HRP blocker was added to each slide and incubated for 15 min at 40°C. All incubations at 40°C were carried out in a humidity control chamber (HybEZ oven, ACDbio). The slides were washed in a washing buffer for 2 min at room temperature, and the last step was repeated with a fresh washing buffer. Signals from other channels were developed similarly. The Opal™ 620 dye FP1495001KT from Akoya Biosciences was assigned to channel 3. Finally, the slides were counterstained with DAPI and stored at 4°C for microscopy.

#### 2.5.6 Confocal microscopy

All slides were scanned using the Zeiss LSM 710 NLO confocal microscope (Zeiss, White Plains, NY). Wavelength absorbance‐emission values were DAPI (410‐449), Adam17 (484‐552), and Aif (599‐670). Using an oil immersion objective, *z*‐stacks of the mPFC were obtained at 63× magnification (Objective i Plan‐Apochromat 63x/1.4 Oil DIC M27; field of view = 25 mm). Additional images were captured using an Andor BC43 Spinning Disk Confocal system (Andor Technology, Belfast) with an oil immersion objective (Plan Apo 60×, NA 1.4; Nikon). Excitation was achieved using 405 nm, 488 nm, 561 nm, and 640 nm lasers in sequence, and emission light was detected by an Andor sCMOS camera (4.2MP; 6.5 µm pixel size).

#### 2.5.7 RNAscope image analysis

The HALO platform (Indica Labs, Albuquerque, NM) with the multiplex fluorescence in situ hybridization (FISH) module was utilized for image analysis. Quantitative gene expression evaluation was performed at a single‐cell resolution. The multiplex FISH module allowed the quantification of RNA FISH probes on a cell‐by‐cell basis. Single cells were identified using the nuclear dye DAPI, and the Adam17 and Aif probes were measured within the cell membrane, presented as spots per cell.

### 2.6 Study 3: Determining the effects of adolescent chronic psychosocial stress and prefrontal cortical TACE/ADAM17 on behavior

Fifty‐two adolescent Lewis rats (postnatal day, PND, 15) were acquired from Charles River Laboratories (Portage, MI). Animals were habituated to housing conditions with 12‐h light/dark phases for at least 1 week before the initiation of the experiments. The rats were weaned at PND22. Rats were matched by sex, body weight, and startle reactivity. Subsequently, the rats were assigned into six groups based on trauma exposure and treatment: (1) Naïve + Unexposed (*n* = 8), (2) Naïve + PSS (*n* = 8), (3) Control + Unexposed (*n* = 12), (4) Control + PSS (*n* = 8); (5) siRNA + Unexposed (*n* = 10), (6) siRNA + PSS (*n* = 13). Unexposed and naïve groups were housed in pairs (same sex and treatment). Naïve rats were used to control for the effects of the surgical manipulation. The data on naïve animals is presented in an earlier iteration of this work (in bioRxiv) and excluded to facilitate data interpretation of the siRNA administration on the stress model. Both sexes were included in each group (half males and half females). The rats were given ad libitum access to water and a purified diet (product no. F7463; Bio‐Serv, Frenchtown, NJ). This diet was used to provide continuity to our studies. Food consumption was monitored, and body weight was measured every week. While classic behavioral readouts were acquired during the light phase, naturalistic behaviors were examined during long periods (24–48 h), encompassing light and dark phases. The study timeline is summarized in Figure 3. Surgical manipulations and siRNA administration methods are detailed above (Study 2).

### 2.7 Automated observation cage behavioral measures

We employed PhenoTyper cages for behavioral assessment through instrumented observation (Noldus Information Technology BV in Wageningen, the Netherlands). These instrumented observation cages are equipped with several key components: (1) a bottom plate resembling a black square arena; (2) four transparent walls, which are replaceable and feature ventilation holes at the top; (3) an illuminated shelter with the capability to be controlled via a hardware control module, enabling automatic lighting when the animal enters the shelter or selecting a specific shelter entrance; (4) a top unit that houses an infrared‐sensitive camera with three arrays of infrared light‐emitting diode (LED) lights; and (5) a range of sensors and stimuli, including adjustable light conditions for establishing a day/night cycle, a single tone for operant conditioning tests, and a white spotlight for examining approach‐avoidance behavior. Instrumented observation cages provide a testing environment that closely resembles the rodents' home cages, allowing the evaluation of various naturalistic behavioral traits. Dark bedding was utilized to enhance visibility. The rats were observed in two separate sessions within these cages: Session 1, spanning 48 h, involving 24 h of acclimation, and Session 2, lasting 24 h. The observations were meticulously recorded and analyzed using EthoVision XT video tracking software, incorporating the Automated Rat Behavior Recognition module developed by Noldus Information Technology. This software enabled the quantification of various behaviors, including grooming, jumping, supported rearing, unsupported rearing, twitching, sniffing, walking, resting, eating, and drinking, with default settings that yield approximately 71% accuracy in detecting individual behaviors (Dam et al., 2013). Behavioral scores were generated exclusively from data collected after the initial 24‐h acclimation period. We also measured eating frequency and duration metrics and interactions with the food hopper using feeding monitors with beam break devices (Noldus).

### 2.8 Acoustic startle reflex (ASR)

The acoustic startle reflex (ASR) experiments were conducted during the light phase using the SR‐LAB acoustic chambers (San Diego Instruments, San Diego, CA, USA). These chambers were equipped with Plexiglas startle enclosures housing piezoelectric transducers and motion sensors to measure startle magnitudes precisely. Before the commencement of the experiments, we meticulously calibrated the intensities of acoustic stimuli and response sensitivities. The experimental sessions commenced with a 5‐min acclimation period, maintaining consistent background noise and light conditions. The background noise level was 55 decibels (dB), while the ambient light conditions were maintained at 400 lux (lx). Following the acclimation period, the rats were subjected to 30 tones, each delivered at a fixed intensity of 105 dB, with a 30‐s intertrial interval. Each acoustic stimulus lasted 20 ms and was presented in a quasi‐random order of trial exposures. Accelerometer readings were collected at 1 ms intervals for a 200 ms period following the initiation of each startle‐inducing acoustic stimulus. The total duration of the experiment was 22 min. All data were meticulously recorded using SR‐LAB startle software. Subsequently, the ASR results were normalized to body weight and then averaged for analysis.

### 2.9 Social Y‐Maze (SYM)

Sociability was assessed during the light phase using a modified Y‐maze and implementing a protocol adapted from Vuillermot et al. (2017). The test was previously used to measure rodent social interactions (Weber‐Stadlbauer et al., 2017). The Social Y‐Maze (Conduct Science, Maze Engineers, Skokie, IL) is a plexiglass Y‐maze with a triangular center (8 cm sides) and three identical arms (50 cm × 9 cm × 10 cm, length × width × height). In this setup, one arm of the apparatus served as the “start arm,” while the other two arms were furnished with rectangular wire mesh cages designed to accommodate live conspecifics or inanimate “dummy objects.” The experimental protocol entailed a single social interaction test trial, omitting prior habituation training. The test animal was initially placed into the start arm and given a 9‐min period to explore the maze freely. During this time, an unfamiliar conspecific of the same sex as the test subject was introduced into one of the rectangular wire mesh cages. Simultaneously, a “dummy object” constructed from multicolored LEGO^®^ pieces (Billund, Denmark) was placed within the other wire mesh cage. Importantly, the placement of conspecifics and “dummy objects” was counterbalanced across arms and the different treatment groups. Between trials, the maze underwent thorough cleaning with 70% ethanol and was allowed to dry. A camera, mounted directly above the maze, recorded each test session, and video recordings were subsequently analyzed using EthoVision XT tracking software developed by Noldus Information Technology.

### 2.10 Multidimensional analysis of rat behavior

Principal component analysis (PCA) was performed to identify group behavioral differences and determine specific behaviors contributing to group variability. The process consisted of normalizing the data, generating principal components (PCs), and performing parallel analysis. Normalization involved scaling the data to a standard normal distribution with a mean and standard deviation of 0 and 1, respectively. Principal components were calculated using GraphPad Prism 9/10 (GraphPad Software, La Jolla, CA, USA). Parallel analysis utilizes Monte Carlo simulations (1000 simulations; Percentile level of 95%) to select PCs with eigenvalues greater than the mean of corresponding PCs at the user‐defined percentile level.

We used *z*‐score calculations to normalize the behavioral data acquired from Ethovision. *Z*‐score normalization is a statistical tool derived by standardizing a group of values by a group average and standard deviation. This allows for a direct comparison between measurements from different scales to a single normalized scale. The following equation was used to calculate the *z*‐scores:

$$z\;=\frac{X-\;\mu }{\sigma },$$

where *X* symbolizes an individual value for an observed parameter; μ and *σ* represent the mean and standard deviation for all animals, respectively. The mean and standard deviation were calculated per behavior using all values regardless of time or group. *Z*‐score values were averaged for each group giving an average *z*‐score value. PCA revealed four distinct behavioral clusters (Figure 3c): (1) ingestive (eating + drinking), (2) exploratory (rearing unsupported + rearing supported + sniffing), (3) ambulatory (walking + twitching + jumping), and (4) maintenance (grooming + resting). Thus, we computed behavioral scores to determine which clusters contributed to the intergroup variability (Guilloux et al., 2011). For example, ingestive behavioral scores were calculated for each subject by averaging eating and drinking *z*‐scores for each rat.

$$\mathrm{Ingestive}\;\mathrm{score}\;=\frac{z_{\mathrm{Eating}}+\;z_{\mathrm{Drinking}}}{\mathrm{Number}\;\mathrm{of}\;\mathrm{ingestive}\;\mathrm{behaviors}}.$$

Individual behavioral scores were averaged within a group to produce a group behavioral score. A comprehensive phenotypic score was obtained by summing all behavioral scores and dividing them by the number of total behavioral scores. Figure S1 shows the data pipeline to acquire a phenotypic score.

$$\mathrm{Phenotypic}\;\mathrm{score}\;=\frac{z_{\mathrm{ingestive}}+\;z_{\mathrm{ambulatory}}+z_{\mathrm{exploratory}}+\;z_{\mathrm{maintenance}}}{\mathrm{Number}\;\mathrm{of}\;\mathrm{behavioral}\;\mathrm{scores}}.$$

Shapiro–Wilk statistical analyses were used to determine sample distribution. The Brown–Forsythe test was used to test for the equality of group variances. Two‐way or three‐way analysis of variance (ANOVA) or mixed analyses were used when appropriate to examine the effect of the intervention (siRNA), stress (psychosocial stress, PSS), time, and interaction between factors on outcome measures. Multiple comparisons were made using Tukey's test. The ROUT method was used to investigate outliers. We used the Pearson's correlation coefficient to examine associations between neuroimaging metrics, TACE/ADAM17, and behavioral outcomes. When appropriate, multiple testing adjustments of the correlations were determined by the false discovery rate (FDR). Differences were considered significant when *p *< .05. The data are shown as the mean ± standard error of the mean.

## 3 RESULTS

### 3.1 Study 1

#### 3.1.1 Psychosocial stress exposure during adolescence attenuates the acoustic startle magnitude and indices of sociability while increasing extracellular water volume and TACE/ADAM17 mRNA levels in the mPFC

The tumor necrosis factor alpha‐converting enzyme/a disintegrin and metalloproteinase 17 (TACE/ADAM17) is critical for the cleavage of different growth factors and inflammatory mediators (Sommer et al., 2019). We reported a model in which supraoptimal TACE/ADAM17 activities may contribute to neuroinflammation and altered brain maturation (Vega‐Torres et al., 2022). This follow‐up study tests the hypothesis that TACE/ADAM17 contributes to behavioral alterations associated with chronic psychosocial stress (PSS). Male and female Lewis rats underwent experimental manipulations and behavioral readouts during critical brain maturational periods.

**FIGURE 1:**
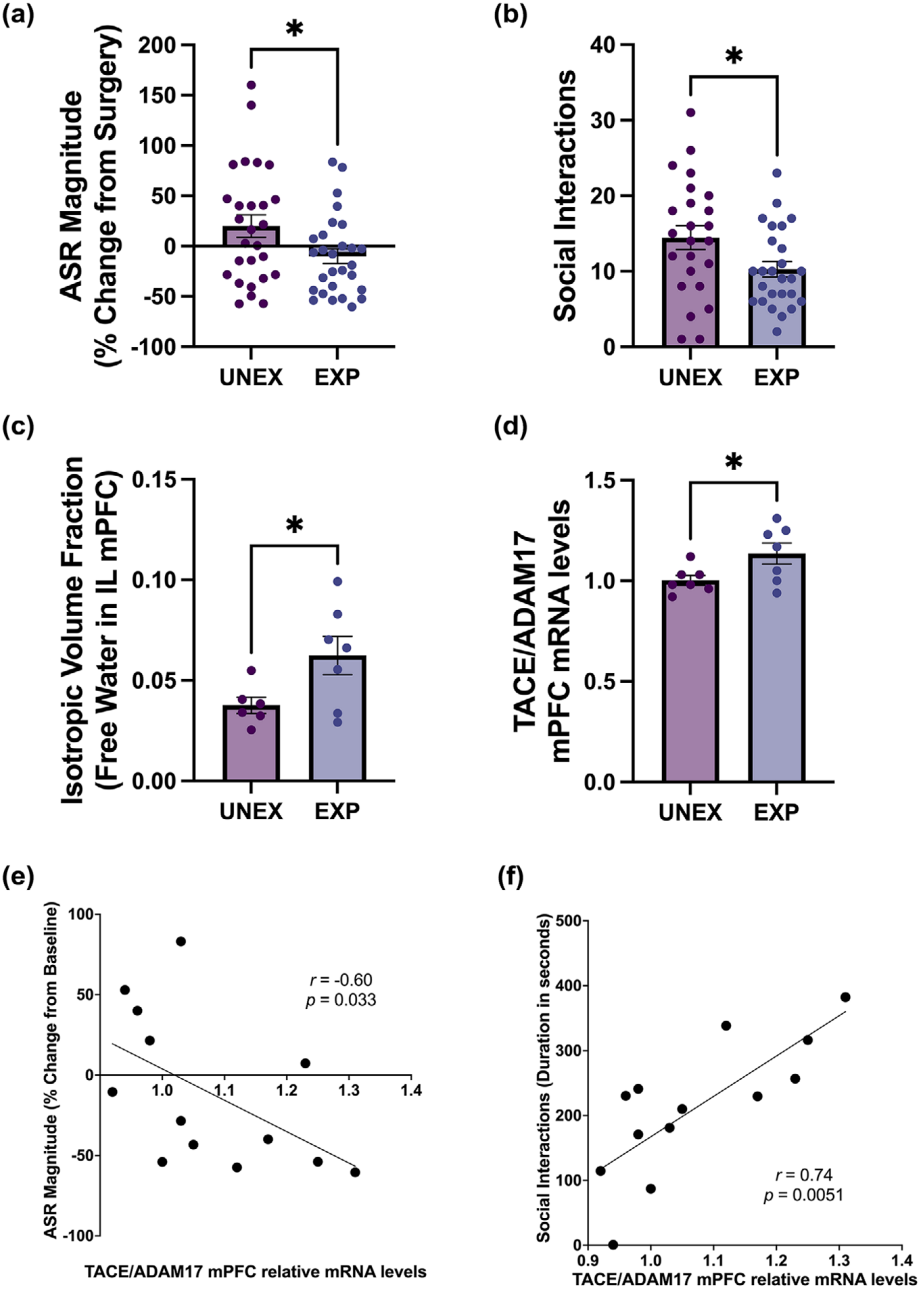
Enduring brain and behavioral consequences of adolescent chronic psychosocial stress in rats. Adolescent male rats underwent chronic psychosocial stress (PSS), behaviors were assessed 1‐week post‐stress, and brains were collected for ex vivo neuroimaging and molecular analyses. (a) Adolescent PSS blunts the magnitude of the acoustic startle reflex (ASR) in male rats (*t*_(53) _= 2.26, *p *= .028). (b) PSS reduces the number of social interactions with a same‐sex conspecific in the social Y‐maze task (*t*_(48) _= 2.26, *p *= .028). (c) PSS increases the isotropic index (unrestricted water diffusion) in the infralimbic cortex as measured with diffusion‐MRI approaches (*t*_(11) _= 2.25, *p *= .046). (d) PSS increases TACE/ADAM17 mRNA levels in the prefrontal cortex (*t*_(12) _= 2.28, *p *= .042). The ASR magnitude (e) and the duration of social interactions (f) were significantly correlated with TACE/ADAM17 mRNA levels in the prefrontal cortex (ASR: Pearson *r* = −0.6, *p* = .033; Social: Pearson *r* = 0.74, *p* = .0051).

We adapted an established ethological rat model that incorporates critical elements of PSS, such as uncontrollability, unpredictability, lack of social support, and re‐experiencing of trauma (Zoladz et al., 2012). In this paradigm, rats are immobilized and exposed to a living predator twice for 1 h, occurring 10 days apart (once at night and then at daytime to model unpredictability and re‐experiencing). Rats undergo a fragmented social structure rearing throughout the experimental timeline. We evaluated the effects of PSS on the acoustic startle reactivity and found that PSS blunted the magnitude of the ASR (*t*_(53) _= 2.26, *p *= .028) (Figure 1a). PSS also reduced the number of social interactions with a same‐sex conspecific (*t*_(48) _= 2.26, *p *= .028) (Figure 1b). We conducted advanced ex vivo diffusion‐MRI analyses and determined the isotropic index to examine whether PSS during adolescence altered freely diffusing, unrestricted water content (0: low isotropy; 1: high isotropy). Analyses revealed that PSS increased the isotropic index relative to controls (*t*_(11) _= 2.25, *p *= .046) (Figure 1c), demonstrating higher free water content, a proxy previously associated with neuroinflammatory processes. We performed qRT‐PCR analysis to evaluate the effect of PSS on TACE/ADAM17 mRNA levels in the mPFC. Results demonstrated a significant increase in TACE/ADAM17 mRNA levels in stressed rats (*t*_(12) _= 2.28, *p *= .042) (Figure 1d). We examined correlations between mRNA levels of TACE/ADAM17 and behaviors to better understand potential implications for TACE/ADAM17 expression in the mPFC. We identified a significant negative correlation between TACE/ADAM17 mRNA levels and ASR magnitude change from baseline (*r* = −0.6; *p* = .033) (Figure 1e). We also found that TACE/ADAM17 mRNA levels in the mPFC were robustly associated with the duration of the interactions with the conspecific in the Social Y‐Maze (*r* = 0.74; *p* = .0051) (Figure 1f).

### 3.2 Study 2

#### 3.2.1 TACE/ADAM17 siRNA administration to the mPFC significantly reduces TACE/ADAM17 mRNA levels without inducing cytotoxicity

**FIGURE 2:**
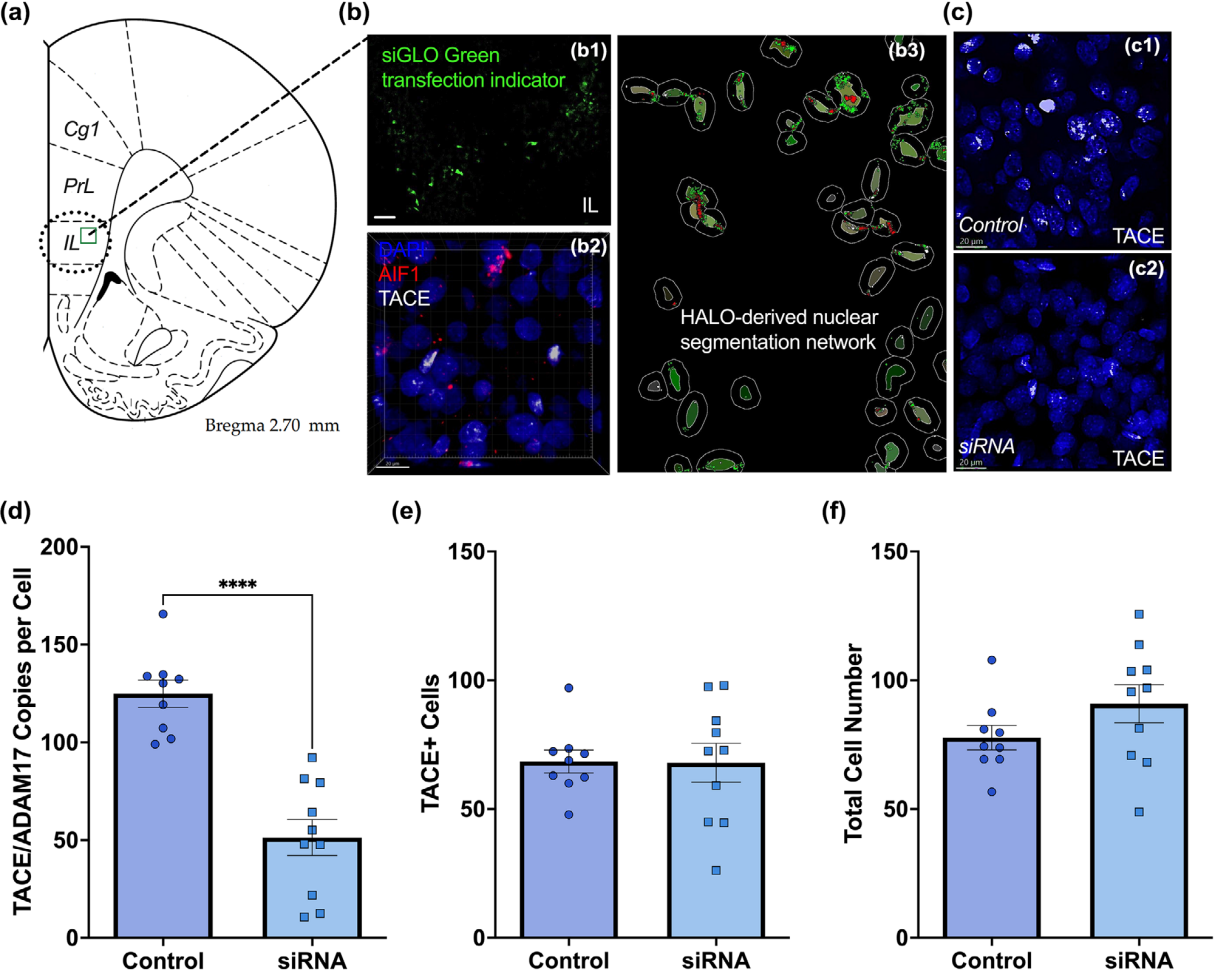
Intracerebral injection of TACE/ADAM17 siRNA significantly decreased TACE/ADAM17 mRNA levels in the mPFC. (a) Illustration from Paxinos and Watson rat brain atlas depicting injection site (infralimbic cortex; IL). (b1) Photomicrograph of rat brain IL injected with siGLO oligos, showing siRNA diffusion (scale bar = 50 micrometers). (b2) Representative photomicrograph of merged RNAScope *z*‐stacks performed to determine TACE/ADAM17 and AIF1 mRNA levels in the mPFC. (b3) The HALO imaging with multiplex fluorescence in situ hybridization (FISH) module showing nuclear segmentation. The HALO platform was used for quantification and analyses. Representative photomicrographs of control (c1) and siRNA (c2) injected brains showing reduced TACE/ADAM17 mRNA levels in the mPFC of siRNA‐treated rats. (d) RNAScope with multiplex FISH analyses confirmed that the TACE/ADAM17 siRNA administration significantly reduced TACE/ADAM17 mRNA levels in the mPFC (*t*_(17) _= 6.282, *p *< .0001). (e) TACE/ADAM17 siRNA administration did not alter the total number of TACE/ADAM17 positive cells per image in the mPFC (*t*_(17) _= 0.055, *p *= .96). Images were acquired using a Zeiss objective (i Plan‐Apochromat 63×/1.4 Oil DIC M27; N.A. = 1.4; fov = 25 mm). (f) TACE/ADAM17 siRNA injections did not alter the total cell number in the mPFC (*t*_(17) _= 1.46, *p *= .16). Scale bars = 20 micrometers. Controls, *n* = 9 rat brains; siRNA = 10 rat brains. ****p < .0001.

Having established that PSS alters behavior and increases prefrontal cortical TACE/ADAM17 mRNA levels, we administered a rat sequence‐specific siRNA to attenuate TACE/ADAM17 expression and examine its functional significance under stress conditions (Figure 2). First, we injected a nontargeting control siRNA to validate siRNA diffusion into mPFC cells (Figure 2a). Intracerebral injection of nontargeting control siRNA allows for assessing the siRNA delivery and uptake by the brain cells. The nontargeting siRNA was labeled with 6‐FAM and visualized with fluorescence microscopy. Our results indicate efficient delivery and uptake of siRNA by mPFC cells, mostly in the infralimbic cortex (Figure 2b1).

Next, we performed an intracerebral injection of siRNA to silence TACE/ADAM17. The duration of siRNA effects can also be influenced by factors such as the efficiency of siRNA delivery, cellular uptake, and intracellular processing. Advanced delivery methods, such as lipid nanoparticles, viral vectors, or proprietary Accell delivery technology, may enhance siRNA stability and prolong its effects compared to simple transfection techniques. An advantage of the Accell passive delivery strategy we employed is its ability to reduce cytotoxic and proinflammatory effects associated with lentiviral or lipid‐based products. This feature allows for prolonged gene knockdown, which is particularly advantageous for identifying chronic effects, as traditional siRNA methods typically provide knockdown lasting only 4–6 days. Notably, a published study employing this approach in rats achieved robust delivery, neuronal incorporation, and target silencing for up to 1 week with a single Accell siRNA injection (Nakajima et al., 2012). We measured TACE/ADAM17 mRNA using a fluorescent RNA in situ hybridization method (Figure 2b2 and 3). As expected, TACE/ADAM17 siRNA significantly decreased TACE/ADAM17 mRNA levels in the mPFC compared to the vehicle‐treated group at 72 h post‐injection (*t*_(17) _= 6.28, *p *< .0001) (Figure 2c and d). This finding corroborates the efficiency of the siRNA in silencing prefrontal cortical TACE/ADAM17 mRNA levels. The number of TACE/ADAM17‐positive cells was unaffected by the intervention (*t*_(17) _= 0.055, *p *= .96) (Figure 2e). Similarly, the total cell number was not affected by the TACE/ADAM17 siRNA administration (*t*_(17) _= 1.46, *p *= .16) (Figure 2f). Together, these data demonstrate that the TACE/ADAM17 siRNA was not toxic to cells.

**FIGURE 3:**
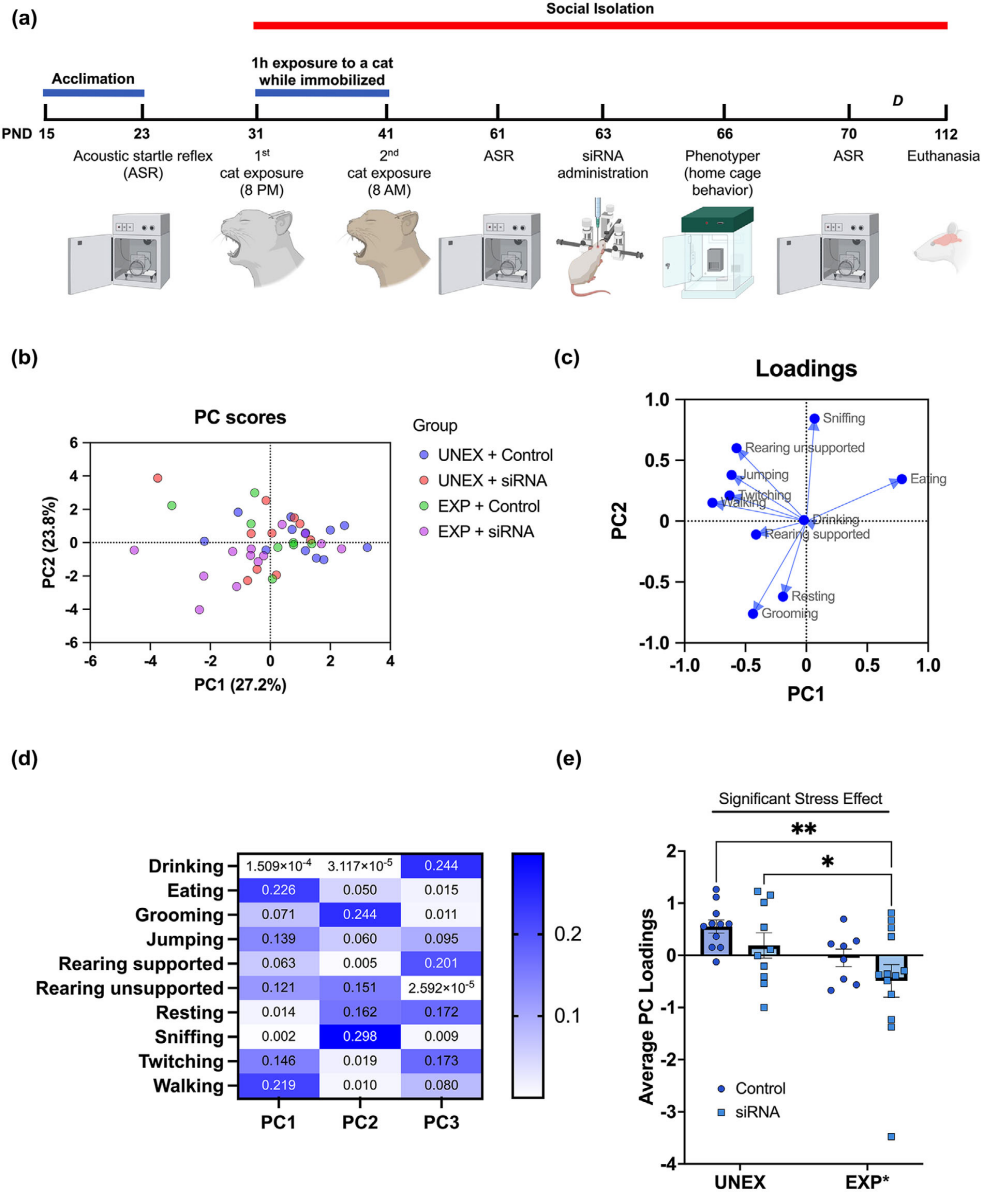
Timeline of experimental procedures, behavioral tests, and outcome measures. (a) Adolescent rats (postnatal day, PND, 23) were matched based on their acoustic startle reflex (ASR) and allocated to one of the four groups: (1) control unexposed (Control UNEX), (2) siRNA unexposed (siRNA UNEX), (3) control exposed (Control EXP), and (4) siRNA exposed (siRNA EXP). The chronic psychosocial stress (PSS) protocol consisted of two exposures to a cat that lasted 1 h each while the animals were immobilized. Exposures were on days 1 (PND31) and 10 (PND41) of the PSS. The animals in the exposure group subsequently underwent social isolation composed of single housing during the experimentation period. ASR measures were collected before the beginning of the PSS protocol (PND23), before (PND61) and after (PND70) siRNA surgeries, and during the final week of behavior (PND107). The siRNA injection was performed on PND63. We evaluated home cage‐like behaviors in the Phenotyper on PND66. Additional long‐term outcomes were examined, including high‐fat food intake and social behaviors (letter D in panel a; see Figure S6). All the rats were euthanized on PND112. (b) Individual Principal Component scores for all animals on a graph of PC1 versus PC2. Each point represents a subject belonging to one of the following groups: UNEX + Control, UNEX + siRNA, EXP + Control, or EXP + siRNA. (c) Loadings plot of PC1 versus PC2 shows behavior‐behavior and behavior‐PC relationships: positive (∼0° difference in direction), negative (∼180°), or no (∼90°) correlation. (d) A heatmap shows the contribution of each behavior to respective principal components. PC1 is characterized primarily by eating, walking, twitching, jumping, and rearing unsupported. For PC2, grooming, sniffing, resting, and rearing contribute the most. For PC3, drinking, rearing supported, resting, and twitching are the behaviors that contribute the most variability. (e) PSS significantly shifts PC loadings, demonstrating that stress alters overall behavioral output in the observation cages (PSS: *F*_(1, 38)_ = 6.67, *p *= .013). UNEX + Control, *n* = 7; UNEX + siRNA, *n* = 10; EXP + Control, *n* = 8; EXP + siRNA, *n* = 13 before outlier testing. *, *p *= .0487; **, *p *= .0027.

### 3.3 Study 3

#### 3.3.1 TACE/ADAM17 siRNA administration influences eating‐related behaviors

Having established the efficacy of the intracerebral siRNA injection to attenuate TACE/ADAM17, we decided to examine whether the intervention altered behavioral proxies related to prefrontal network integrity. Considering the fundamental roles of prefrontal networks on top‐down behavior control, we monitored the TACE/ADAM17 siRNA injection effects on ethologically relevant behaviors (Table 1). Optimal gene knockdown at the mRNA level is usually reached at 48–72 h after Accell™ siRNA delivery; thus, we commenced to measure behavioral outcomes after this period. Figure 3a describes the experimental timeline and procedures for Study 3. We used observation home cages and automated behavior recognition tracking software (Dam et al., 2013) to determine behavioral profiles in four rat groups: (1) PSS unexposed + control intracerebral injection (UNEX + control), (2) PSS unexposed + siRNA intracerebral injection (UNEX + siRNA), (3) PSS exposed + control intracerebral injection (EXP + control), and (4) PSS exposed + siRNA intracerebral injection (EXP + siRNA). Monitoring behaviors in this naturalistic environment over long periods provides complex and ethologically relevant information that may reflect changes in conserved endophenotypes (Figure S1). The data presented in Figure S2 provide an overview of behavioral patterns observed within the PhenoTyper cages, shedding light on the influence of stress and prefrontal cortical TACE/ADAM17 siRNA injection on these behaviors. We observed distinct impacts on behavioral parameters, including differences in the magnitude and timing of these changes. The arrows indicate the most prominent differences in behavior observed among the groups. In the observation cages, variations in resting, sniffing, twitching, and walking behaviors were notably pronounced during the initial 24 h (see Figure S2). Conversely, changes in grooming, jumping, and rearing behaviors became more apparent on the second day of observation (see Figure S2). For a comprehensive overview of the statistical analysis, including *F* statistics for each behavior, please refer to Table 2. Overall, the siRNA treatment and stress appeared to interact with time, influencing the trajectories of behavioral patterns observed in the observation cage. Our data suggest a dynamic interplay between these factors in shaping behavior. These findings underscore the importance of considering the temporal dimension when assessing the effects of siRNA treatment and stress on behavioral outcomes.

**TABLE 1:** List and definition of naturalistic behaviors acquired and examined.

Variable	Description
*Drinking*	The subject licks at the spout of the water bottle.
*Eating*	The subject eats at the feeder or from the floor or is eating while holding food in front paws.
*Grooming*	The subject groom snout, head, fur or genitals. Includes scratching and licking of paws during a grooming session.
*Jumping*	The subject moves quickly forward with both hind limbs at the same time. Jumping interrupts other states like Walking.
*Rearing unsupported*	The subject stands in an upright posture, with front paws not in contact with any object. Includes the rise and descend.
*Rearing supported*	The subject stands in an upright posture, leaning with front paws against the cage‐ wall. Includes the rise and descend.
*Resting*	The subject rests with hardly any moving, either sits or is lying down. Includes sleeping. Apparently, no interest in the environment.
*Sniffing*	The subject makes slight movements of the head, possibly with slight, discontinuous body displacement. Includes sniffing the air, the wall, the floor and other objects.
*Twitching*	The subject makes sudden and short movements of the body or head. Includes body shake and head shake. Twitching is scored as a point event with no duration and does not interrupt behavior states.
*Walking*	The subject moves to another place, and hind legs move as well.

**TABLE 2:** Detailed summary of home‐cage behavior statistics.

*Source of Variation*	Time	Stress (PSS)	Treatment (siRNA)	Time × Stress	Time × Treatment	Stress × Treatment	Time × Stress × Treatment
**Behavior**							
Eating	***F* (8.189, 286.6) = 30.06,** ***p *< .0001**	*F* (1, 35) = 1.608, *p *= .2131	*F* (1, 35) = 1.802, *p *= .1881	*F* (22, 770) = 1.186, *p *= .2524	***F* (22, 770) = 2.193,** ***p *= .0013**	*F* (1, 35) = 0.2563, *p *= .6159	*F* (22, 770) = 1.164, *p *= .2732
Drinking	***F* (8, 280) = 3.628,** ***p *= .0005**	*F* (1, 35) = 0.02078, *p *= .8862	*F* (1, 35) = 0.01519, *p *= .9026	*F* (22, 770) = 1.416, *p *= .976	*F* (22, 770) = 0.8601, *p *= .6493	*F* (1, 35) = 0.8092, *p *= .3745	*F* (22, 770) = 0.5005, *p *= .9737
Grooming	***F* (9.431, 330.1) = 6.202,** ***p *< .0001**	*F* (1, 35) = 0.2285, *p *= .6356	*F* (1, 35) = 1.344, *p *= .2542	***F* (22, 770) = 2.016,** ***p *= .0039**	*F* (22, 770) = 0.3818, *p *= .9957	*F* (1, 35) = 0.003921, *p *= .9504	***F* (22, 770) = 1.904,** ***p *= .0075**
Jumping	***F* (8.860, 310.1) = 5.391,** ***p *< .0001**	*F* (1, 35) = 0.1094, *p *= .7428	*F* (1, 35) = 0.1001, *p *= .7536	*F* (22, 770) = 1.410, *p *= .1000	*F* (22, 770) = 1.270, *p *= .1822	*F* (1, 35) = 0.6472, *p *= .4265	*F* (22, 770) = 1.080, *p *= .3626
Rearing Supported	***F* (7.793, 272.7) = 11.25,** ***p *< .0001**	*F* (1, 35) = 0.3572, *p *= .5539	*F* (1, 35) = 0.8999, *p *= .3493	*F* (22, 770) = 1.061, *p *= .3850	*F* (22, 770) = 1.102, *p *= .3377	*F* (1, 35) = 0.08142, *p *= .7771	*F* (22, 770) = 0.08142, *p *= .8112
Rearing Unsupported	***F* (8.045, 273.5) = 14.85,** ***p *< .0001**	*F* (1, 34) = 3.125, *p *= .0861	*F* (1, 34) = 0.01740, *p *= .8958	*F* (22, 748) = 1.124, *p *= .3140	*F* (22, 748) = 0.5928, *p *= .9303	*F* (1, 34) = 0.7948, *p *= .3789	*F* (22, 748) = 1.307, *p *= .1569
Resting	***F* (4.303, 150.6) = 2.480,** *p *= .**0423**	*F* (1, 35) = 1.364, *p *= .2508	*F* (1, 35) = 0.07903, *p *= .7803	***F* (22, 770) = 1.664,** ***p *= .0287**	*F* (22, 770) = 0.9032, *p *= .5911	*F* (1, 35) = 0.02077, *p *= .8862	*F* (22, 770) = 0.9987, *p *= .4633
Sniffing	***F* (8.102, 283.6) = 7.490,** ***p *< .0001**	*F* (1, 35) = 1.855, *p *= .1819	*F* (1, 35) = 1.931, *p *= .1734	*F* (22, 770) = 0.6686, *p *= .8723	*F* (22, 770) = 0.6662, *p *= .8745	*F* (1, 35) = 0.8108, *p *= .3740	*F* (22, 770) = 1.022, *p *= .4338
Twitching	***F* (9.002, 315.1) = 10.89,** ***p *< .0001**	*F* (1, 35) = 1.752, *p *= .1942	*F* (1, 35) = 0.1846, *p *= .6701	***F* (22, 770) = 1.670,** ***p *= .0279**	*F* (22, 770) = 1.236, *p *= .2090	*F* (1, 35) = 1.484, *p *= .2312	*F* (22, 770) = 1.404, *p *= .1028
Walking	***F* (7.992, 279.7) = 5.875,** ***p *< .0001**	*F* (1, 35) = 0.003129, *p *= .9557	*F* (1, 35) = 0.1007, *p *= .7529	*F* (22, 770) = 0.6237, *p *= .9092	*F* (22, 770) = 1.098, *p *= .3421	*F* (1, 35) = 0.01095, *p *= .9172	*F* (22, 770) = 0.8403, *p *= .6757

Probabilistic behavioral data from the first complete PhenoTyper session was analyzed via three‐way ANOVA. Bold denotes factors and interactions showing significant effects (***p* < .05**). PSS, psychosocial stress; siRNA, TACE/ADAM17 silencing RNA intracerebral administration.

We found that TACE/ADAM17 siRNA administration had opposite effects on food intake depending on PSS exposure (stress: *F*_(1, 36) _= 0.068, *p *= .80; treatment: *F*_(1, 36) _= 4.08, *p *= .051; interaction: *F*_(1, 36) _= 5.57, *p *= .024) (Figure S3a). We also found that the PSS rats that received the siRNA displayed increased ambulation during the light cycle (time: *F*_(4.15, 149.1)_ = 39.97, *p* < .0001; stress: *F*_(1, 790)_ = 3.16, *p* = .08; treatment: *F*_(1, 790)_ = 0.16, *p* = .69; time × stress: *F*_(22, 790)_ = 0.70, *p* = .84; time × treatment: *F*_(22, 790)_ = 0.83, *p* = .69; stress × treatment: *F*_(1, 790)_ = 6.42, *p* = .012; time × stress × treatment *F*_(22, 790)_ = 2.067, *p* = .0028) (Figure S3b). Similarly, the PSS rats that received the siRNA exhibited a higher probability of performing eating behaviors (time: *F*_(8.189, 286.6)_ = 30.06, *p* < .0001; stress: *F*_(1, 35)_ = 1.61, *p* = .21; treatment: *F*_(1, 35)_ = 1.80, *p* = .19; time × stress: *F*_(22, 770)_ = 1.19, *p* = .25; time × treatment: *F*_(22, 770)_ = 2.19, *p* = .0013; stress × treatment: *F*_(1, 35)_ = 0.26, *p* = .62; time × stress × treatment: *F*_(22, 770)_ = 1.16, *p* = .27) (Figure S2b) and spent more time in the food zone during the light cycle when compared to controls (time: *F*_(4, 175)_ = 0.78, *p* = .54; stress: *F*_(1, 175)_ = 7.82, *p* = .0057; treatment: *F*_(1, 175)_ = 8.51, *p* = .0040; time × stress: *F*_(4, 175)_ = 0.96, *p* = .43; time × treatment: *F*_(4, 175)_ = 0.81, *p* = .52; stress × treatment: *F*_(1, 175)_ = 7.87, *p* = .0056; time × stress × treatment: *F*_(4, 175) _= 0.94, *p* = .44) (Figure S3c). This effect was not observed during the dark cycle (time: *F*_(4, 175)_ = 2.5, *p* = .041; stress: *F*_(1, 175)_ = 0.08, *p* = .78; treatment: *F*_(1, 175) _= 0.083, *p* = .77; time × stress: *F*_(4, 175)_ = 1.86, *p* = .12; time × treatment: *F*_(4, 175)_ = 0.16, *p* = .96; stress × treatment: *F*_(4, 175)_ = 0.0078, *p* = .93; time × stress × treatment: *F*_(4, 175)_ = 0.90, *p* = .46) (Figure S3d). Together, these results present new evidence that prefrontal cortical TACE/ADAM17 may influence eating behaviors, energy metabolism, and circadian rhythms under stress conditions (Gelling et al., 2008).

#### 3.3.2 Psychosocial stress leads to alterations in startle reactivity and naturalistic behaviors

**FIGURE 4:**
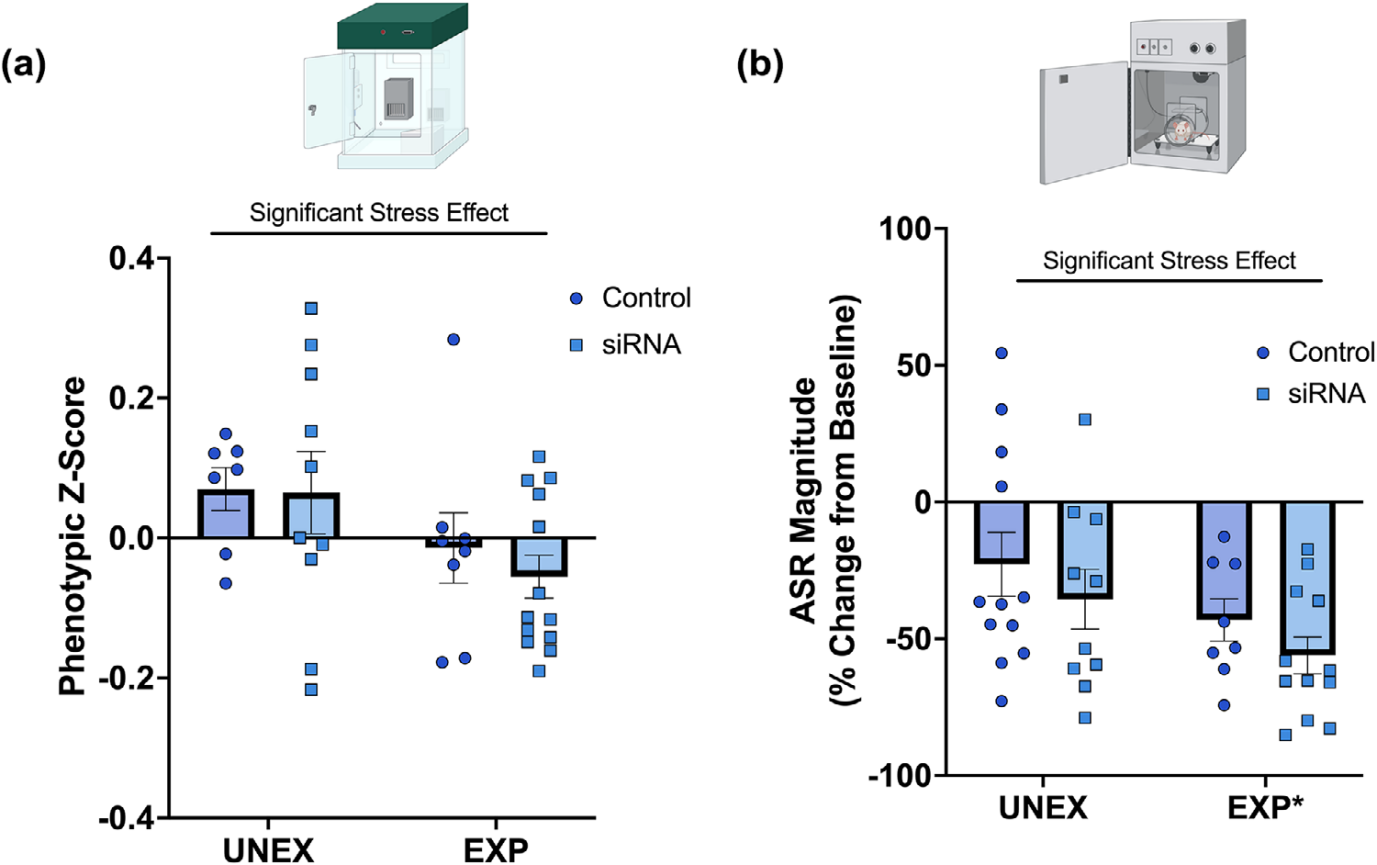
Chronic adolescent psychosocial stress leads to behavioral alterations. (a) Phenotypic scores from the first testing session at PND66. Behavioral probabilistic data from the second consecutive day inside the PhenoTyper observation cages was derived from Ethovision XT's Automated Behavior Recognition. Raw probabilistic data were transformed via PCA and *z*‐normalization to generate an integrated phenotypic score. PSS significantly shifts the phenotypic score (*F*_(1, 34)_ = 4.69, *p* = .038). (b) Exposure to PSS during adolescence dampened the ASR magnitude, confirming Study 1 in male rats (*F*_(1, 38)_ = 4.26, *p *= .046). UNEX + Control, *n* = 7; UNEX + siRNA, *n* = 10; EXP + Control, *n* = 8; EXP + siRNA, *n* = 13 before outlier testing.

We used principal component analysis (PCA) and *z*‐normalization to reduce dimensionality and identify distinct behavioral profiles. Figure S1 details the sequence of behavioral data processing. We computed the behavioral probability for each behavior across time during the testing session in the observation cages. Figure 3a depicts the timeline and experimental procedures. Probabilistic data was used to generate principal components and reduce the dimensionality of the datasets. The principal component analysis (PCA) produced three factors that explained 68.8% of the variance between groups (Figure 3b and Figure S4). Figure 3c and d describes loadings and their contributions to the variability. *Component 1* (27.2% of variance) was mainly loaded by eating (contribution = 0.23) and walking (contribution = 0.22). *Component 2* (23.8% of variance) was explained by sniffing (contribution = 0.30) and grooming (contribution = 0.24). *Component 3* (17.8% of variance) was primarily loaded by drinking (contribution = 0.24) and supported rearing (contribution = 0.20). ANOVA of the scores for components 1, 2, and 3 from the PCA showed significant effects for PSS (stress: *F*_(1, 38)_ = 6.67, *p* = .014), demonstrating that rats exposed to PSS exhibit a behavioral shift in overall home‐cage behaviors (treatment: *F*_(1, 38)_ = 2.63, *p* = .11; interaction: *F*_(1, 38)_ = 0.022, *p* = .88) (Figure 3e). By delving deeper into the PCA loadings, we identified four main behavioral clusters: (1) ingestive (eating and drinking), (2) maintenance (grooming and resting), (3) ambulatory (jumping, twitching, and walking), (4) exploratory (rearing supported, rearing unsupported, and sniffing). We normalized the data using z‐scores to understand better whether PSS altered behavioral clusters in the PhenoTyper. *Z*‐scores transform data into a common scale, making comparing and combining data from testing sessions and integrating potential sex‐dependent variability easier. This method also improves data visualization and facilitates data interpretation. Probabilistic data and corresponding *z*‐scores are summarized in the heatmaps provided in Figure S5. We found that PSS during adolescence resulted in a significant shift in the combined “phenotypic *z*‐score,” encompassing the four main behavioral domains measured in the PhenoTyper (stress: *F*_(1, 34)_ = 4.98, *p* = .032; treatment: *F*_(1, 34)_ = 0.26, *p* = .62; interaction: *F*_(1, 34)_ = 0.15, *p* = .70) (Figure 4a).

A primary outcome measure of startle reactivity is the acoustic startle reflex (ASR) magnitude, a direct measure of attention and arousal states. We have used ASR measures to match animals into groups based on emotionality and determine the short‐ and long‐term impact of stressors and dietary manipulation in rats (Kalyan‐Masih et al., 2016; Vega‐Torres et al., 2022; Vega‐Torres et al., 2018; Vega‐Torres et al., 2020). In agreement with other studies, we found that PSS exposure decreased the magnitude of the ASR (stress: *F*_(1, 38)_ = 4.26, *p* = .046; treatment: *F*_(1, 38)_ = 1.68, *p* = .20; interaction: *F*_(1, 38)_ = 0.0001, *p* = .99) (Figure 4b).

#### 3.3.3 Long‐term effects of TACE/ADAM17 siRNA administration and PSS on behavior

We conducted secondary experiments to determine the long‐term effects of prefrontal cortical TACE/ADAM17 siRNA administration and PSS on behavior (Figure S6a). The cumulative eating duration (Figure S6b), ASR magnitude and latency (Figure S6c–e), duration of social interactions (Figure S6f), and total distance traveled (Figure S6g) were comparable between groups (*p* > .05 for all factors; *F* stats are included in the figure legend). Data normalization with *z*‐scores showed that the effect of stress on social behavior differed depending on whether the rats received TACE/ADAM17 siRNA or not (significant interaction effect: *F*_(1, 38)_ = 4.93, *p* = .032; additional *F* stats are included in the figure legend) (Figure S6h). This suggests that the influence of adolescent stress on social behavior is not uniform across all conditions but somewhat varies depending on TACE/ADAM17 expression in the prefrontal cortex. This highlights the complex interplay between these two variables in shaping social behavior.

**FIGURE 5:**
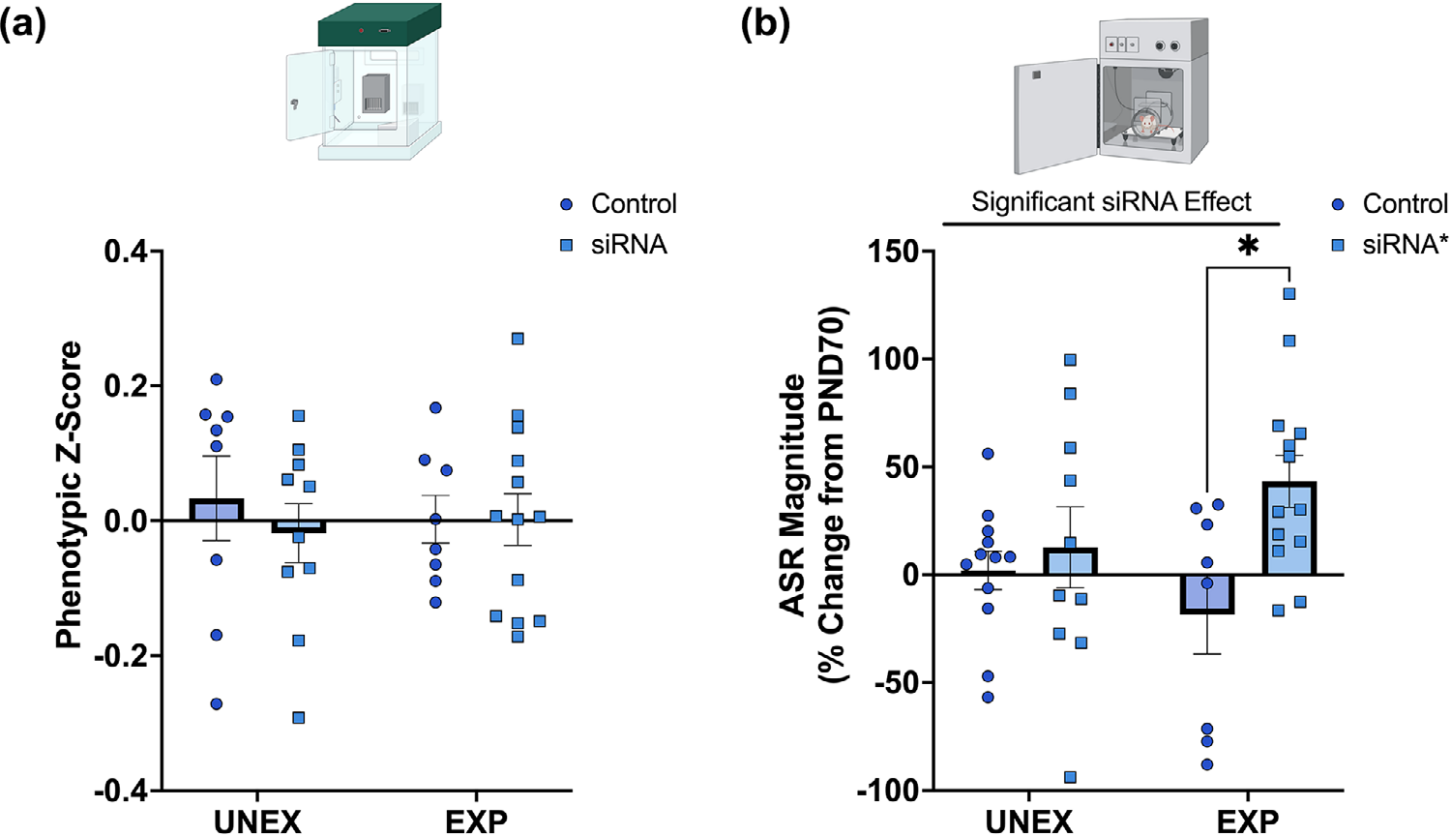
Long‐term effects of intracerebral TACE/ADAM17 siRNA administration and psychosocial stress on naturalistic behaviors and acoustic startle reflex. (a) The phenotypic score was comparable between groups at PND100. (b) Rats that received the TACE/ADAM17 siRNA exhibited increased ASR magnitudes at PND107 relative to PND70 (*F*_(1, 39) _= 6.3, *p *= .0157). *p = .016. UNEX + Control, *n* = 7; UNEX + siRNA, *n* = 10; EXP + Control, *n* = 8; EXP + siRNA, *n* = 13 before outlier testing.

The phenotypic *z*‐score was similar between groups (stress: *F*_(1, 35)_ = 0.014, *p* = .90; treatment: *F*_(1, 35)_ = 0.32, *p* = .58; interaction: *F*_(1, 35)_ = 0.31, *p* = .58) (Figure 5a). Interestingly, although the final ASR magnitudes and latencies were similar between groups (Figure S6c), siRNA rats exhibited increased percent change in ASR relative to controls (% ASR magnitude from PND70 to PND107) (stress: *F*_(1, 39)_ = 0.12, *p* = .72; treatment: *F*_(1, 39)_ = 6.38, *p* = .016; interaction: *F*_(1, 39)_ = 3.16, *p* = .083) (Figure 5b). This finding suggests that TACE/ADAM17 may play a role in the prefrontal networks that modulate this reflex and its habituation. We also examined binge eating‐like behaviors in a subcohort of animals. Both UNEX and EXP rats were combined into a single group after observing no statistically significant difference in WD intake between the two groups (stress: *F*_(1, 37)_ = 0.98, *p* = .33; diet: *F*_(2, 37)_ = 17.88, *p* < .0001; interaction: *F*_(2, 37)_ = 0.68, *p* = .51). We found that TACE/ADAM17 siRNA rats given intermittent access to an obesogenic diet consumed more food than vehicle controls at 2.5 h after reintroducing the obesogenic diet (treatment *F* stats *p* < .05; *F* stats provided in the figure legend) (Figure S7a). This effect was only transient, as rats exhibited similar food intake during the 24‐h period after the obesogenic diet was re‐introduced (*p* > .05; *F* stats provided in the figure legend) (Figure S7b and c). Although TACE/ADAM17 mRNA levels were not directly assessed at the conclusion of Study 3, it is important to note that the duration of Accell siRNA effects has been demonstrated to extend beyond that of other passive siRNAs. It remains conceivable that downregulating TACE/ADAM17 expression during critical subacute phases of traumatic stress (e.g., fear memory consolidation) could mitigate or prevent the behavioral complications associated with stress. It is also plausible that TACE/ADAM17 silencing altered the learning and memory processes during the initial exposure to the testing conditions, potentially influencing subsequent exposures. Hence, the concept of a therapeutic window may offer insight into a potential explanation of the observed long‐term effects.

#### 3.3.4 TACE/ADAM17 silencing reduces the expression of the allograft inflammatory factor 1

Our research findings indicate that environments conducive to obesity, characterized by chronic stress and access to unhealthy diets, induce significant immunometabolic alterations in rats (Ontiveros‐Angel et al., 2023). Moreover, evidence suggests a potential association between these alterations and the activity of TACE/ADAM17 (Vega‐Torres et al., 2022). TACE/ADAM17 is critical in regulating microglial responses to proinflammatory insults by cleavage and release of survival, proliferative, and inflammatory mediators (Feuerbach et al., 2017; Sommer et al., 2019; Vidal et al., 2013; Wei et al., 2015). Nevertheless, the role of this protease in healthy states remains relatively unknown. We examined the TACE/ADAM17 siRNA effects on the allograft inflammatory factor 1 (AIF1). AIF1 (also known as IBA1) is expressed in microglia, the immune cells of the central nervous system. It plays critical roles in microglial activation, morphology regulation, and involvement in neuroinflammation, particularly in neurodegenerative diseases like Alzheimer's and Parkinson's. AIF1 has potential as a biomarker for neuroinflammation and may be a target for therapeutic interventions in neurological disorders. As expected, AIF1 colocalized with TACE/ADAM17+ cells (Figure S8). We found that knocking down TACE/ADAM17 mRNA with siRNA significantly decreased the Aif1 expression level in the mPFC at 72 h post‐injection (*t*_(17)_ = 8.78, *p *< .0001) (Figure S8). The percentage of AIF+ cells that expressed TACE/ADAM17 was also reduced in the mPFC of siRNA‐treated rats relative to controls (*t*_(17)_ = 3.50, *p *= .0027) (Figure S8). Although Accell siRNA demonstrates a tendency for preferential incorporation into neurons (Nakajima et al., 2012), our findings underscore a role of TACE/ADAM17 in maintaining the homeostasis of microglial cells under normal physiological conditions.

## 4 DISCUSSION

This research delved into the repercussions of chronic stress experienced during adolescence on a spectrum of behavioral aspects. We examined the role of prefrontal cortical TACE/ADAM17 in modulating the startle reflex, social interactions, and naturalistic behaviors within a home‐cage‐like environment. The results unveiled that chronic stress dampens startle reactivity while inducing alterations in naturalistic behaviors, including grooming, resting, and twitching. Interestingly, we present new evidence that prefrontal cortical TACE/ADAM17 may regulate aspects of food intake.

The medial prefrontal cortex (mPFC) plays a pivotal role in modulating stress responses and regulating the associated behaviors and physiological changes (Arnsten, 2009). The mPFC exerts top‐down control over the hypothalamic‐pituitary‐adrenal (HPA) axis, a central player in the body's stress response system. Through its extensive connections with other brain regions, including the amygdala and the hippocampus, the mPFC integrates emotional and contextual information to fine tune the stress response. One of the key functions of the mPFC in stress regulation is the inhibition of the HPA axis activity. The mPFC sends inhibitory signals to the paraventricular nucleus of the hypothalamus, reducing the release of corticotropin‐releasing hormone (CRH) and subsequently attenuating the release of glucocorticoids, such as cortisol in humans and corticosterone in rodents (Herman, 2013). Dysregulation of this inhibitory control within the mPFC is associated with heightened stress responses. It contributes to the pathophysiology of stress‐related disorders like post‐traumatic stress disorder (PTSD) and major depressive disorder (MDD). Additionally, the mPFC is involved in the extinction of fear responses, a process crucial for overcoming traumatic memories, highlighting its significance in stress resilience and recovery (Sotres‐Bayon et al., 2004). Understanding the intricate mechanisms through which the mPFC modulates stress responses is essential for developing targeted interventions for stress‐related disorders.

The anatomy and function of the medial prefrontal cortex (mPFC) in rodents have been subject to ongoing controversy and debate. While the mPFC is commonly subdivided into regions such as the infralimbic cortex (IL) and prelimbic cortex (PL), the precise boundaries and functional distinctions between these subregions remain a topic of contention. Given the considerable anatomical and functional differences between species, there is debate regarding the extent to which rodent mPFC homologs correspond to human prefrontal areas (Laubach et al., 2018; Preuss, 1995). Some researchers argue that the mPFC in rodents may not directly mirror the complex cognitive and emotional functions attributed to the human prefrontal cortex, making cross‐species comparisons challenging (Uylings et al., 2003). The controversy surrounding the mPFC in rodents underscores the need for continued research to elucidate its anatomical organization and functional roles, potentially leading to a more nuanced understanding of its contributions to behavior and cognition.

The PhenoTyper is a sophisticated behavioral monitoring system widely utilized to assess various aspects of behavior in rats. This system employs video tracking and can be used with advanced software algorithms to precisely quantify and analyze behaviors in a controlled laboratory environment (Dam et al., 2013). Researchers have employed the PhenoTyper to investigate a range of behaviors, including locomotion, exploration, anxiety‐related behaviors, social interactions, and more (Grieco et al., 2021; Pham et al., 2009; Prevot et al., 2019; Spruijt & DeVisser, 2006). Furthermore, it has been instrumental in studies examining the effects of drugs, genetic manipulations, and environmental factors on rat behavior, making it a valuable tool for advancing our understanding of neurobiology and behavior in preclinical research. By capturing detailed data on movement, position, and interactions, the PhenoTyper allowed us to assess behavior over extended periods objectively, reducing observer bias and enhancing the reproducibility of results. Furthermore, using PCA‐based *z*‐normalization lowered the variance of phenotypic measures and improved the reliability of probabilistic data. In support of prior studies, we found that chronic psychosocial stress during adolescence profoundly influences various naturalistic behavioral domains, including ingestive, ambulation, social, and exploratory behaviors in rats, particularly during early adulthood. Several studies have provided valuable insights into these stress‐induced alterations in rodent behavior. Stress‐induced changes in eating behavior are exemplified in a study by Dallman et al. (2003), where chronic stress led to altered food intake patterns and increased preference for palatable, high‐energy foods in rats. Several lines of evidence support that severe stress usually results in hypophagia in rodents, which is supported by the findings presented in this study. However, in agreement with Dallman et al. (2003) and others, stressed animals exhibited more binge eating‐like behaviors when presented with intermittent access to a high‐fat diet. This shift in dietary preference toward comfort foods may have implications for developing obesity and metabolic disorders under chronic stress conditions, particularly in humans. Concurrently, changes in ambulation, indicative of locomotor activity, have been reported in stressed rats. Longstanding studies demonstrated that exposure to mild stressors significantly altered the diurnal and circadian rhythms of locomotor activity (Gorka et al., 1996). Similarly, we found that stressed rats exhibited more ambulation during the light cycle. Rats are nocturnal animals, and their activity levels are typically higher during the dark cycle. Increased ambulation during the light cycle could be linked to disruptions in circadian rhythms or alterations in activity patterns that can contribute to increased eating. While the results of studies are controversial, stress also impacts exploratory behaviors in rats. Rats exposed to a chronic unpredictable stress paradigm exhibited reduced rearing behaviors in an observation home cage (Eraslan et al., 2023). Our findings revealed that stress influenced rearing behaviors in the observation home cage (higher probability of exhibiting supported rearing and lower probability of exhibiting unsupported rearing). In agreement with other studies, we observed reduced maintenance behaviors (self‐grooming and resting) in rats exposed to chronic adolescent stress. It is recognized that maintenance behaviors like grooming can follow a U‐shaped pattern. It usually happens naturally when the animal is at a low state of arousal, serving as a maintenance behavior to keep clean. As the arousal level increases to a moderate range, self‐grooming becomes more prolonged, and the pattern of grooming may change. This shift can be seen as a form of displacement activity, reflecting the animal's response to increased arousal. However, when stress levels reach a high point, leading to responses like freezing, fighting, or fleeing, self‐grooming tends to be inhibited or reduced. In such intense stress situations, rodents seem to prioritize survival‐related actions over grooming behaviors (Fernández‐Teruel & Estanislau, 2016; Kalueff et al., 2016; Song et al., 2016). These findings collectively highlight the multifaceted effects of stress on rat behavior and underscore the importance of further research to elucidate the underlying neurobiological mechanisms driving these changes. However, there is an urgent need to understand the molecular players influencing behavior in individuals exposed to chronic stress during adolescence.

In agreement with prior studies, chronic psychosocial stress during adolescence suppressed the magnitude of the acoustic startle reflex (ASR) (Adamec et al., 2006; Beck et al., 2008; Conti & Printz, 2003). As described in previous work from our group, the decreased startle reactivity could indicate changes in sensory thresholds for evoking ASR, motor impairments, and peripheral mechanisms. One such mechanism involves inflammation, which has been found to suppress startle responses by promoting interleukin‐1 production in immune‐sensitive Lewis rats (Beck & Servatius, 2006). Consequently, it is plausible that the stress, by priming the immune system, blunts the ASR. This notion is supported by our recent work demonstrating increased interleukin‐1 levels in Lewis rats exposed to PSS (Ontiveros‐Angel et al., 2023).

Chronic psychosocial stress has been associated with a broad range of neuropathophysiological effects, including neuroinflammatory responses. While the exact mechanisms and causality are still subjects of ongoing research, our data demonstrate that chronic psychosocial stress increases the uniform diffusion of extracellular water content in the prefrontal cortex, a marker previously associated with cell shrinkage, aging, and inflammation (Merluzzi et al., 2015; Syková & Nicholson, 2008). In support, recent research in transgenic rats with Alzheimer's‐like pathology reported changes in isotropic index over time associated with the degree of inflammation (Fick et al., 2017). Previous studies have also shown higher isotropic indices in the brains of individuals with autism, multiple sclerosis, and Parkinson's, all involving neuroinflammation as a significant factor in the disease process (Andica et al., 2021; Hagiwara et al., 2019; Kamagata et al., 2017). Our prior studies validate that obesogenic conditions induce inflammatory responses associated with altered brain structure and behavior (Ontiveros‐Angel et al., 2023). AIF‐1, or allograft inflammatory factor 1, is a protein associated with inflammation and immune responses. It is also known as IBA1 (Ionized Calcium‐Binding Adapter Molecule 1) and is primarily expressed in macrophages and microglia, immune cells in the central nervous system. AIF‐1 plays a role in the activation and migration of macrophages and microglia, and its expression is often upregulated in response to inflammatory stimuli. It has been used as a marker to identify and study the activation of these immune cells in various pathological conditions, including stress‐induced neuroinflammation in animal models (Cohen et al., 2016; Ontiveros‐Angel et al., 2023). Although AIF‐1 is not an exclusive or primary marker of inflammation, it has served as a valuable tool for investigating microglial activities. The study revealed that the modulation of TACE resulted in a significant reduction in the expression of this marker, supporting the potential involvement of TACE in microglial processes (Feuerbach et al., 2017; Sommer et al., 2019). Understanding the underlying pathways involved in these potential proinflammatory changes is crucial for developing interventions and treatments to mitigate the negative consequences of chronic stress on brain maturation and well‐being.

Metalloproteases regulate vital cellular signals, providing crucial proteolytic shedding activities (Blobel, 2005; Weber & Saftig, 2012; Yang et al., 2006). During nervous system development, a disintegrin and metalloproteinases (ADAMs) significantly influence neuronal differentiation, proliferation, migration, and axonal myelination (Yang et al., 2006). The ADAM17 (also known as the tumor necrosis factor converting enzyme or TACE) has important roles in activating neural cell adhesion and neurite outgrowth, processes that are critical during brain maturation (Black et al., 1997; Kalus et al., 2006; Maretzky et al., 2005). TACE/ADAM17 regulates amyloid precursor protein (APP), which is associated with neuronal migration and synaptic connectivity during development (Allinson et al., 2003; Löffler & Huber, 1992; Young‐Pearse et al., 2007). TACE/ADAM17 is pivotal in regulating crucial developmental signaling pathways associated with the epidermal growth factor receptor (EGF‐R) (Blobel, 2005). In agreement with prior studies from our group (Vega‐Torres et al., 2022), TACE/ADAM17 upregulation under obesogenic conditions may contribute to EGF‐R family dysregulation, possibly contributing to altered brain structural changes and emotionality. Emerging evidence proposes that TACE/ADAM17 may also influence metabolism. Gelling et al. (2008) reported that, despite typical food intake, TACE/ADAM17 adult homozygous TACE/ADAM17‐deficient mice exhibit hypermetabolism and a lean phenotype. Given the ubiquitous expression profile of TACE/ADAM17, it is difficult to determine which organ(s) contributed to this effect, but the authors proposed that sympathetic outflow may be enhanced in this mouse model. Our findings demonstrating increased ambulation and eating partly support a heightened sympathetic outflow. Interestingly, while stress‐exposed rats exhibited altered ambulation and spent more time in the food zones in response to siRNA administration, unexposed rats that received the siRNA demonstrated increased food intake relative to controls. This divergence in siRNA responses may reflect competing influences between anxiety‐induced exploratory behavior, novelty‐seeking behavior, and homeostatic regulation. It is reasonable to hypothesize that TACE/ADAM17 abrogation under physiological conditions may disrupt mPFC homeostasis and induce a phenotype resembling stress‐induced changes in food intake. On the other hand, attenuating TACE/ADAM17 under proinflammatory stress conditions led to increased ambulation and time spent in the feeding zone, particularly during the light cycle. These findings offer insights into the intricate pathways governing stress‐induced behavioral alterations and highlight a role of TACE/ADAM17 in modulating these responses.

Proinflammatory conditions are known to alter TACE/ADAM17 expression. We demonstrated that consuming a high‐saturated fat obesogenic diet during adolescence increased hippocampal TACE/ADAM17 protein levels (Vega‐Torres et al., 2022). Consuming the obesogenic diet significantly increased neuroinflammatory mediators, particularly the tumor necrosis factor‐alpha (TNF‐α). These molecular alterations were associated with alterations in hippocampal volumetric parameters (Vega‐Torres et al., 2022). Considering the crucial role of TNF‐α as an essential substrate for TACE/ADAM17, responsible for mediating inflammatory and developmental processes, it is plausible that TACE/ADAM17 may exert regulatory control over adolescent brain maturation (Black et al., 1997; Idriss & Naismith, 2000; Yang et al., 2006).

This study encompasses certain limitations that warrant further investigation. To gain a more comprehensive understanding, it is imperative to ascertain the spatiotemporal expression patterns of mPFC TACE/ADAM17 and the associated cytokine profiles, shedding light on the underlying mechanisms. Our findings did not examine whether the silencing effects of TACE/ADAM17 are mediated by microglia, influenced by alterations in the extracellular environment through intermediary factors, or in response to neuronal activities and synaptic plasticity. Therefore, it is imperative to undertake experiments aimed at disentangling the relative contributions of neural phenotypes to the observed effects. Future research endeavors should employ genetic or pharmacological tools allowing sustained blockade to distinguish between the chronic and acute roles of TACE/ADAM17 in response to stress. While the study included female subjects, it is essential to replicate critical experiments with larger sample numbers and perform statistical analyses to discern potential sex‐specific differences. Caution should be exercised in interpreting the results, particularly when extrapolating to human conditions, as anatomical disparities exist between rats and humans, especially concerning the PFC.

In summary, this study integrates behavioral, neuroimaging, and molecular data to demonstrate the long‐lasting impact of adolescent chronic stress on a wide range of naturalistic behaviors, prefrontal cortical tissue integrity, and TACE/ADAM17 mRNA levels. By delving into the complexities of home‐cage continuous and longitudinal behaviors and integrative *z*‐normalization, this study contributes to understanding the broader and dynamic impact of psychosocial stress exposure during adolescence in rats. Furthermore, this study identifies prefrontal cortical TACE/ADAM17 as a potential modulator of stress‐related locomotor activity and eating. Understanding how adolescent stress shapes prefrontal cortical maturational trajectories may help to identify measures to prevent and alleviate the burden of mental illness in the emerging adult population.

## AUTHOR CONTRIBUTIONS

**Fransua Sharafeddin**: Conceptualization; investigation; writing—original draft; methodology; writing—review and editing; formal analysis; data curation. **Julio Sierra**: Methodology; validation; visualization; writing—review and editing; software; formal analysis; data curation. **Mina Ghaly**: Data curation; formal analysis; validation. **Timothy B. Simon**: Writing—original draft; investigation; visualization; validation; methodology; software; formal analysis; data curation. **Perla Ontiveros‐Ángel**: Investigation; writing—original draft; methodology. **Brandon Edelbach**: Formal analysis; software. **Marcelo Febo**: Investigation; methodology; software; formal analysis; data curation. **Jennifer Labus**: Validation; formal analysis; software. **Johnny D. Figueroa**: Conceptualization; investigation; funding acquisition; writing—original draft; validation; visualization; writing—review and editing; software; formal analysis; project administration; data curation; supervision; resources.

## CONFLICT OF INTEREST STATEMENT

All authors report no financial interests or potential conflicts of interest.

### PEER REVIEW

The peer review history for this article is available at https://publons.com/publon/10.1002/brb3.3482.

## Supporting information

Supplementary Information

Figure S1

Figure S2

Figure S3

Figure S4

Figure S5

Figure S6

Figure S7

Figure S8

## Data Availability

In addition to the data presented in the supplementary materials, supportive datasets are available from the corresponding author upon reasonable request.
