# Supplementary material for: Role of the prefrontal cortical protease TACE/ADAM17 in neurobehavioral responses to chronic stress during adolescence
Source: Brain Behav. 2024 May 7;14(5):e3482. doi: 10.1002/brb3.3482 (PMC11077197; doi:10.1002/brb3.3482)
Supplement: Supplementary file 1 — Supplementary Information [file BRB3-14-e3482-s006.docx]

**Supplementary Methods**

**Neuroimaging Procedures**

Here, we incorporated methodologies reported by our collaborator and co-author, Dr. Marcelo Febo (Colon-Perez *et al.* 2019)**.** The preservation of the anatomical integrity of rat brains, encapsulated within the cranium, was accomplished in accordance with previously reported techniques (Wilkes et al., 2020). The cerebral tissue was immersed in FC-40 (Fluorinert™) within a 50 ml polypropylene conical tube, allowing for an equilibration period of no less than 12 hours at room temperature prior to the commencement of scanning. A custom plastic fixture was thoughtfully introduced into the tube to stabilize the specimen physically, thereby mitigating motion artifacts during the application of gradient pulsations. High-resolution brain imaging was conducted using an 11.1 Tesla/40 cm bore MRI scanner (Magnex Scientific Ltd., Oxford, UK) equipped with a Resonance Research Inc. gradient set (RRI BFG-240/120-S6), boasting a maximum gradient strength of 1000 mT/m at 325 Amps, and a 200 µs risetime. A Bruker Paravision 6.01 console (Bruker BioSpin, Billerica, MA) managed scanner control, in harmony with established methodologies (Sahara *et al.* 2014). For both excitation and signal reception, a 3.2 x 7.6 cm quadrature birdcage resonator tuned to 470.7 MHz (1H resonance) was diligently employed.

**Diffusion MRI Acquisition**

The acquisition of diffusion-weighted images followed a comprehensive protocol, involving a 4-shot, 2-shell spin echo planar diffusion imaging (DTI EPI) sequence within Bruker Paravision. Parameters for this acquisition included a repetition time (TR) of 4 seconds, echo time (TE) of 19 ms, a number of averages (NA) set at 4, a gradient duration (δ) of 3 ms, a diffusion time (Δ) of 8 ms, encompassing 77 images featuring 3 unique diffusion weightings. These consisted of eight images with b=0, 23 images with b=600 s/mm2, and 46 images with b=1200 s/mm2. Furthermore, a navigator signal was judiciously employed by the Bruker reconstruction software to enhance signal stability during the 4-shot EPI sequence. To suppress extraneous signals, image saturation bands were strategically positioned adjacent to, as well as below, the brain during the image acquisition phase. The resulting images encompassed 1602 voxels per slice across 50 slices, with a voxel size of 0.15 mm2 and a slice thickness of 0.5 mm.

**Diffusion MRI Processing**

Processing of the acquired diffusion MRI scans was conducted employing the resources available within the FMRIB Software Library (FSL) toolkit, as detailed by Smith *et al.* (Smith *et al.* 2004). To rectify minor motion artifacts encountered during image acquisition, an eddy correction procedure was diligently applied. Subsequently, gradient files were aligned in accordance with the motion correction vectors to ensure data accuracy and reliability. Following the eddy correction phase, tensor element reconstruction ensued, accompanied by the estimation of the first, second, and third eigenvectors and eigenvalues, denoted as λ1, λ2, and λ3, respectively. Notably, λ1 represents axial diffusivity, while the average of λ2 and λ3 furnishes values indicative of radial diffusivity. These computations were executed utilizing a weighted least squares regression method, implemented via the DTIFIT tool within the FSL software suite, following the approach outlined by Behrens *et al.* (Behrens *et al.* 2003). Consequently, this process yielded distinct image datasets encapsulating mean diffusivity (MD), axial diffusivity (AD), radial diffusivity (RD), and Fractional Anisotropy (FA), each bearing unique significance in the characterization of diffusion patterns within the examined biological specimens.

The NODDI (Neurite Orientation Dispersion and Density Imaging) processing pipeline employs a multi-compartment model to dissect the diffusion signal. This model encompasses an isotropic fraction representing freely diffusing and hindered water molecules, such as those found in cerebral spinal fluid. Additionally, it incorporates an anisotropic model that delineates an intracellular fraction composed of zero-radius infinite cylinders, exhibiting elevated diffusion coefficients along the principal axis exclusively. Furthermore, it accommodates an extracellular fraction characterized by non-zero diffusion perpendicular to the principal axis. This component enriches the tissue model by providing morphological insights into the extracellular milieu enveloping axons and dendrites, including neurons and non-neuronal cell bodies, as indicated by Zhang *et al.* (Zhang *et al.* 2012). After diffusion MRI scans, data underwent analysis utilizing the NODDI model within the Accelerated Microstructure Imaging via Convex Optimization (AMICO) framework, which expedites the model fitting process, executed on a standard Linux PC running Python software, as described by Daducci *et al.*(Daducci *et al.* 2014). The estimation of the intracellular volume fraction (zero-radius cylinders, often referred to as "sticks") entails the utilization of a Bingham-Watson series, a statistical shape analysis technique elucidated by Maier-Hein *et al.* (Maier-Hein *et al.* 2015) and Tariq *et al.* (Tariq *et al.* 2016). The outcomes of this fitting procedure provide a quantifiable index of the degree of orientation dispersion within the so-called neurites. For parameter fitting, we assumed an ex vivo intrinsic diffusivity of 0.6 µm²/ms and an isotropic diffusivity of 2.0 µm²/ms, in accordance with Grussu *et al.* (Grussu *et al.* 2017). The comprehensive model fitting process yields maps detailing the intracellular volume fraction (ICVF, denoting the relative concentration of zero-radius cylinders modeling neurites), orientation dispersion (ODI, with values ranging from 0 for no dispersion, as observed in highly organized parallel fiber bundles, to a maximum of 1 for the highest degree of dispersion, akin to cerebral cortical grey matter), and the isotropic free water fraction (ISO, spanning from 0 denoting low isotropy to 1 signifying maximum isotropy), following the approach delineated by Grussu *et al.* (Grussu *et al.* 2017).

**Image Normalization and Rat Brain Parcellation**

Primary regions of interest (ROIs) were scrutinized to facilitate the investigation of DTI and NODDI scalar metrics and the normalized Jacobian matrix values reflective of structural disparities among the experimental groups, primary regions of interest (ROIs) were scrutinized. The delineation of B0 scan masks demarcating the boundaries of rat brains was executed utilizing MATLAB, with Three-Dimensional Pulsed Coupled Neural Networks (PCNN3D) (Chou *et al.* 2011) serving as the foundation. To ensure precise alignment, subsequent manual adjustments were performed using ITKSNAP (Yushkevich *et al.* 2006). Subsequently, B0 images were registered to a parcellated rat brain template, as previously detailed by Kenkel *et al.* (Kenkel *et al.* 2016). Linear registration to the template was accomplished employing the FSL linear registration tool (FLIRT) (Jenkinson *et al.* 2002), employing a correlation ratio search cost, complete 180-degree search space, 12 degrees of freedom, and trilinear interpolation. The resultant linear registration outputs underwent nonlinear warping to the template space via Advanced Normalization Tools (ANTs) (Klein *et al.* 2009). The ensuing deformation field images generated Jacobian determinant maps, serving as an evaluative metric for the quality of subject-to-atlas registration and quantifying regional structural distinctions. Subsequently, B0-to-atlas linear and nonlinear transformation matrices were applied to scalar maps. All pertinent data were extracted and summarized utilizing Microsoft Excel (Redmond, WA, USA) and subjected to analysis employing GraphPad Prism version 9.4.1.681 for Windows (GraphPad Software, San Diego, California, USA, [www.graphpad.com](http://www.graphpad.com/)).

**SUPPLEMENTAL FIGURES**

**Supplemental Figure 1. Phenotyper home cage monitoring system and data analysis pipelines.** **(A)** The Phenotyper home-cage monitoring apparatus (left panel) and arena settings (right panel) illustrate zone parcellation. The arena settings show an example of user-defined zones, like a shelter zone, food zone, and drinking zone, specified in Ethovision XT software. **(B)** The Principal Component Analysis (PCA) pipeline shows the automated behaviors recognized and acquired during Phenotyper testing. PCA was used to reduce the dimensionality of the data in the form of principal components (PCs). Individual PC scores are plotted on a graph of PC1 vs PC2 (not actual data). **(C)** Z-score pipeline used during data analysis to determine behavior scores. The diagram exemplifies the protocol to determine an ingestive score and, ultimately, a phenotypic score: **(1)** raw data is collected for all behaviors (shown in B), **(2)** individual subject z-scores are calculated, **(3)** behavior scores are computed by averaging individual z-scores from clustered behaviors into a single behavior score for each individual, and **(4)** the four behavior scores (ingestive, ambulatory, exploratory, and maintenance) are averaged into a phenotypic score for each individual, and further averaged into a final group phenotypic score.

**Supplemental Figure 2. Averaged raw probabilistic data of automated behaviors in the PhenoTyper home cages.** Rats were single-housed in PhenoTyper observation cages for 48 h. For each behavior, probabilistic data was binned in 2 h periods. Averaged probabilities for **(A)** drinking, **(B)** eating, **(C)** grooming, **(D)** jumping, **(E)** rearing supported, **(F)** rearing unsupported, **(G)** resting, **(H)** sniffing, **(I)** twitching, **(J)** walking illustrate distinctive behavioral patterns during the first session in the PhenoTyper (T1). Arrows denote the time of day when behavioral differences between groups were more apparent. Note that some differences were more evident early (first day in the home cages, *habituation*; G-H) while others appeared later (second day in the home cages, *testing*; A-F). Refer to **Table 1** for definitions of each behavior and **Table 2** for details of F statistics. Control Unexposed, n = 12; Control Exposed, n = 8; siRNA Unexposed, n = 10; siRNA Exposed, n = 13.

**Supplemental Figure 3. Prefrontal cortical TACE/ADAM17 siRNA alters eating and ambulatory activity. (A)** TACE/ADAM17 siRNA administration to the medial prefrontal cortex influences food intake in the observation home cage. Analyses demonstrate contrasting TACE/ADAM17 administration effects that depend on psychosocial stress (PSS) exposure (interaction: *F* _(1, 36)_ = 5.57, *p* = 0.024). **(B)** PSS and TACE/ADAM17 siRNA administration interact to impact ambulation in the observation home cages. PSS rats that received the TACE/ADAM17 siRNA exhibited increased ambulation during the light cycle (stress x treatment: *F* _(1, 790)_ = 6.42, *p* = 0.012). (C) Stress and TACE/ADAM17 siRNA influence the time spent in the feeding zone (stress: *F* _(1, 175)_ = 7.82, *p* =0.0057; treatment: *F* _(1, 175)_ = 8.51, *p* = 0.0040; stress x treatment: *F* _(1, 175)_ = 7.87, *p* = 0.0056). Note that PSS rats that received the siRNA exhibited more time in the food zone during the light cycle when compared to controls. **(D)** PSS and TACE/ADAM siRNA administration failed to alter the time spent in the food zone during the dark phase (*p* > 0.05 for all factors). Control Unexposed, n = 12; Control Exposed, n = 8; siRNA Unexposed, n = 10; siRNA Exposed, n = 13.

**Supplemental Figure 4. Proportion of the total variance explained by each Principal Component.** Behavioral probability data was used to generate Principal Components. **(A)** Timepoint 1 **(T1)** PCs from behavioral data acquired 4 days after siRNA injection surgery. The first three PCs explain **68.8%** of the total variance in the data. The variance explained by principal components PC1, PC2, and PC3 are 27.2%, 23.8%, 17.8%, respectively. **(B)** Proportion of variance explained by each PC for data acquired during the final testing session **(T2)**. The first two principal components explain **57.4%** of the total variance in the data. The variance explained by principal components PC1 and PC2 are 36.9% and 20.5%, respectively.

**Supplemental Figure 5. Automated behavior recognition analyses identify changes in the probability of home-cage behaviors. (A, B)** **Timepoint 1 (T1)** heatmaps showing averaged raw probabilistic data (A) and z-scores of behavioral probabilities (B) for each group (UNEX + Control, UNEX + siRNA, EXP + Control, and EXP + siRNA). **(C, D) Timepoint 2 (T2)** heatmaps showing averaged raw probabilistic data (C) and z-scores of behavioral probabilities (D) for each group (UNEX + Control, UNEX + siRNA, EXP + Control, and EXP + siRNA). Control Unexposed, n = 12; Control Exposed, n = 8; siRNA Unexposed, n = 10; siRNA Exposed, n = 13.

**Supplemental Figure 6. Long-term effects of psychosocial stress and TACE/ADAM17 siRNA administration on behavior. (A)** Timeline of experimental procedures, behavioral tests, and outcome measures. **(B)** The experimental conditions did not significantly affect the eating behavior in the PhenoTyper home cages (stress: *F* _(1, 35)_ = 0.21, *p* = 0.65; treatment: *F* _(1, 35)_ = 0.087, *p* = 0.77; interaction: *F* _(1, 35)_ = 2.79, *p* = 0.10). **(C, D)** Analyses of the acoustic startle reflex (ASR) magnitude (C; peak startle response that occurs during each trial) and latency (D; time from stimulus to the peak startle response) did not show significant effects (for ASR magnitude, stress: *F* _(1, 39)_ = 0.16, *p* = 0.69; treatment: *F* _(1, 39)_ = 1.55, *p* = 0.22; interaction: *F* _(1, 39)_ = 0.81, *p* = 0.37; and for ASR latency, stress: *F* _(1, 39)_ = 1.26, *p* = 0.26; treatment: *F* _(1, 39)_ = 0.11, *p* = 0.74; interaction: *F* _(1, 39)_ = 1.02, *p* = 0.32). **(E)** Combined z-scores for ASR magnitude and latency reveal no significant impact of PSS and TACE/ADAM17 siRNA administration on ASR metrics (stress: *F* _(1, 39)_ = 0.18, *p* = 0.67; treatment: *F* _(1, 39)_ = 0.27, *p* = 0.60; interaction: *F* _(1, 39)_ = 0.0052, *p* = 0.94). **(F)** The time spent interacting with same-sex conspecific was similar between groups (expressed as a percentage of the total time; 9 min test) (stress: *F* _(1, 38)_ = 0.043, *p* = 0.84; treatment: *F* _(1, 38)_ = 0.40, *p* = 0.53; interaction: *F* _(1, 38)_ = 1.63, *p* = 0.21). **(G)** The total distance traveled in the social Y maze was similar between groups (stress: *F* _(1, 39)_ = 0.091, *p* = 0.77; treatment: *F* _(1, 39)_ = 0.58, *p* = 0.45; interaction: *F* _(1, 39)_ = 1.87, *p* = 0.18). **(H)** Z-score data normalization revealed a significant interaction effect (stress: *F* _(1, 38)_ = 0.10, *p* = 0.75; treatment: *F* _(1, 38)_ = 0.21, *p* = 0.65; interaction: *F* _(1, 38)_ = 4.93, *p* = 0.032). Control Unexposed, n = 12; Control Exposed, n = 8; siRNA Unexposed, n = 10; siRNA Exposed, n = 13.

**Supplemental Figure 7. Food intake alterations in rats exposed to an intermittent obesogenic high-fat diet.** A subgroup of rats was introduced to a Western-like obesogenic diet for 48 h used in our prior studies (Vega-Torres *et al.* 2018, 2022). The rats were re-introduced to the diet once a week for three consecutive weeks to generate a well-established model of binge eating (Czyzyk *et al.* 2010; Maske *et al.* 2020). **(A)** Weight-normalized food consumed during 2.5 h following the third experimental cycle. As expected, WD-INT rats consumed more food than CD and WD rats (Diet: *F* _(2, 34)_ = 29.96, *p* < 0.0001; Treatment: *F* _(1, 34)_ = 4.27, *p* = 0.046; Interaction: *F* _(2, 34)_ = 5.01, *p* = 0.012). TACE/ADAM17 siRNA administration increased binge eating-like behavior relative to vehicle controls (*p*=0.0091, difference: -0.0097, 95% CI of difference: -0.018 to -0.0018). **(B)** Weight-normalized food consumed during the 24-h period in cycle 3. Diet: *F* _(2, 37)_ = 17.74, *p* < 0.0001; Treatment: *F* _(1, 37)_ = 0.47, *p* = 0.50; Interaction: *F* _(2, 37)_ = 2.25, *p* = 0.12). Twenty-four h food intake was similar between WD-INT siRNA and control groups, confirming the binge eating phenotype at 2.5 h (*p* = 0.63). **(C)** The total number of nose pokes inside the food monitor was similar between groups. Diet: *F* _(2, 36)_ = 1.74, *p* = 0.19; Treatment: *F* _(1, 36)_ = 0.53, *p* = 0.50; Interaction: *F* _(2, 36)_ = 0.52, *p* = 0.60). CD, control diet; WD, Western-like high-fat diet, WD-INT, WD intermittent access. WD-INT control injection, n = 8; WD-INT siRNA injection, n = 8. **, adjusted *p* = 0.002.

**Supplemental Figure 8. TACE/ADAM17 siRNA intracerebral injection attenuates the expression of a crucial neuroinflammation biomarker in the mPFC. (A)** Representative RNAScope photomicrographs demonstrating allograft inflammatory factor 1 (AIF1; also known as ionized calcium-binding adapter molecule 1 or Iba-1) expressing cells that co-express TACE/ADAM17 mRNA. Representative sections from control-treated rat brain **(A1)** and TACE/ADAM17 siRNA-treated rat brain **(A2)** show decreased TACE/ADAM17 and AIF mRNA levels in siRNA-treated animals. **(B)** TACE/ADAM17 siRNA significantly decreased the AIF1 mRNA levels in the mPFC. **(C)** The percentage of AIF+ cells that expressed TACE/ADAM17 mRNA was also reduced in the mPFC of siRNA-treated rats. Scale bars = 20 micrometers. Controls, n = 9 rat brains; siRNA = 10 rat brains. **, p<0.01; ****, p<0.0001.

**SUPPLEMENTAL REFERENCES**

Behrens, T.E.J., Woolrich, M.W., Jenkinson, M., Johansen‐Berg, H., Nunes, R.G., Clare, S., Matthews, P.M., Brady, J.M. & Smith, S.M. (2003) Characterization and propagation of uncertainty in diffusion‐weighted MR imaging. *Magnet Reson Med* **50**, 1077–1088.

Chou, N., Wu, J., Bingren, J.B., Qiu, A. & Chuang, K.-H. (2011) Robust Automatic Rodent Brain Extraction Using 3-D Pulse-Coupled Neural Networks (PCNN). *Ieee T Image Process* **20**, 2554–2564.

Colon-Perez, L.M., Ibanez, K.R., Suarez, M., Torroella, K., Acuna, K., Ofori, E., Levites, Y., Vaillancourt, D.E., Golde, T.E., Chakrabarty, P. & Febo, M. (2019) Neurite orientation dispersion and density imaging reveals white matter and hippocampal microstructure changes produced by Interleukin-6 in the TgCRND8 mouse model of amyloidosis. *Neuroimage* **202**, 116138.

Czyzyk, T.A., Sahr, A.E. & Statnick, M.A. (2010) A Model of Binge‐Like Eating Behavior in Mice That Does Not Require Food Deprivation or Stress. *Obesity* **18**, 1710–1717.

Daducci, A., Canales-Rodríguez, E.J., Zhang, H., Dyrby, T.B., Alexander, D.C. & Thiran, J.-P. (2014) Accelerated Microstructure Imaging via Convex Optimization (AMICO) from diffusion MRI data. *Neuroimage* **105**, 32–44.

Grussu, F., Schneider, T., Tur, C., Yates, R.L., Tachrount, M., Ianuş, A., Yiannakas, M.C., Newcombe, J., Zhang, H., Alexander, D.C., DeLuca, G.C. & Wheeler‐Kingshott, C.A.M.G. (2017) Neurite dispersion: a new marker of multiple sclerosis spinal cord pathology? *Ann Clin Transl Neur* **4**, 663–679.

Jenkinson, M., Bannister, P., Brady, M. & Smith, S. (2002) Improved Optimization for the Robust and Accurate Linear Registration and Motion Correction of Brain Images. *Neuroimage* **17**, 825–841.

Kenkel, W.M., Yee, J.R., Moore, K., Madularu, D., Kulkarni, P., Gamber, K., Nedelman, M. & Ferris, C.F. (2016) Functional magnetic resonance imaging in awake transgenic fragile X rats: evidence of dysregulation in reward processing in the mesolimbic/habenular neural circuit. *Transl Psychiat* **6**, e763–e763.

Klein, A., Andersson, J., Ardekani, B.A., Ashburner, J., Avants, B., Chiang, M.-C., Christensen, G.E., Collins, D.L., Gee, J., Hellier, P., Song, J.H., Jenkinson, M., Lepage, C., Rueckert, D., Thompson, P., Vercauteren, T., Woods, R.P., Mann, J.J. & Parsey, R.V. (2009) Evaluation of 14 nonlinear deformation algorithms applied to human brain MRI registration. *Neuroimage* **46**, 786–802.

Maier-Hein, K.H., Westin, C.-F., Shenton, M.E., Weiner, M.W., Raj, A., Thomann, P., Kikinis, R., Stieltjes, B. & Pasternak, O. (2015) Widespread white matter degeneration preceding the onset of dementia. *Alzheimer’s Dementia* **11**, 485-493.e2.

Maske, C.B., Coiduras, I.I., Ondriezek, Z.E., Terrill, S.J. & Williams, D.L. (2020) Intermittent High‐Fat Diet Intake Reduces Sensitivity to Intragastric Nutrient Infusion and Exogenous Amylin in Female Rats. *Obesity* **28**, 942–952.

Sahara, N., Perez, P.D., Lin, W.-L., Dickson, D.W., Ren, Y., Zeng, H., Lewis, J. & Febo, M. (2014) Age-related decline in white matter integrity in a mouse model of tauopathy: an in vivo diffusion tensor magnetic resonance imaging study. *Neurobiol Aging* **35**, 1364–1374.

Smith, S.M., Jenkinson, M., Woolrich, M.W., Beckmann, C.F., Behrens, T.E.J., Johansen-Berg, H., Bannister, P.R., Luca, M.D., Drobnjak, I., Flitney, D.E., Niazy, R.K., Saunders, J., Vickers, J., Zhang, Y., Stefano, N.D., Brady, J.M. & Matthews, P.M. (2004) Advances in functional and structural MR image analysis and implementation as FSL. *Neuroimage* **23 Suppl 1**, S208-19.

Tariq, M., Schneider, T., Alexander, D.C., Wheeler-Kingshott, C.A.G. & Zhang, H. (2016) Bingham–NODDI: Mapping anisotropic orientation dispersion of neurites using diffusion MRI. *Neuroimage* **133**, 207–223.

Vega-Torres, J.D., Haddad, E., Lee, J.B., Kalyan-Masih, P., George, W.I.M., Pérez, L.L., Vázquez, D.M.P., Torres, Y.A., Santana, J.M.S., Obenaus, A. & Figueroa, J.D. (2018) Exposure to an obesogenic diet during adolescence leads to abnormal maturation of neural and behavioral substrates underpinning fear and anxiety. *Brain Behav Immun*.

Vega-Torres, J.D., Ontiveros-Angel, P., Terrones, E., Stuffle, E.C., Solak, S., Tyner, E., Oropeza, M., Peña, I. dela, Obenaus, A., Ford, B.D. & Figueroa, J.D. (2022) Short-term exposure to an obesogenic diet during adolescence elicits anxiety-related behavior and neuroinflammation: modulatory effects of exogenous neuregulin-1. *Transl Psychiat* **12**, 83.

Yushkevich, P.A., Piven, J., Hazlett, H.C., Smith, R.G., Ho, S., Gee, J.C. & Gerig, G. (2006) User-guided 3D active contour segmentation of anatomical structures: Significantly improved efficiency and reliability. *Neuroimage* **31**, 1116–1128.

Zhang, H., Schneider, T., Wheeler-Kingshott, C.A. & Alexander, D.C. (2012) NODDI: Practical in vivo neurite orientation dispersion and density imaging of the human brain. *Neuroimage* **61**, 1000–1016.
