# Supplementary figures and images for: Role of the prefrontal cortical protease TACE/ADAM17 in neurobehavioral responses to chronic stress during adolescence
Source: Brain Behav. 2024 May 7;14(5):e3482. doi: 10.1002/brb3.3482 (PMC11077197; doi:10.1002/brb3.3482)

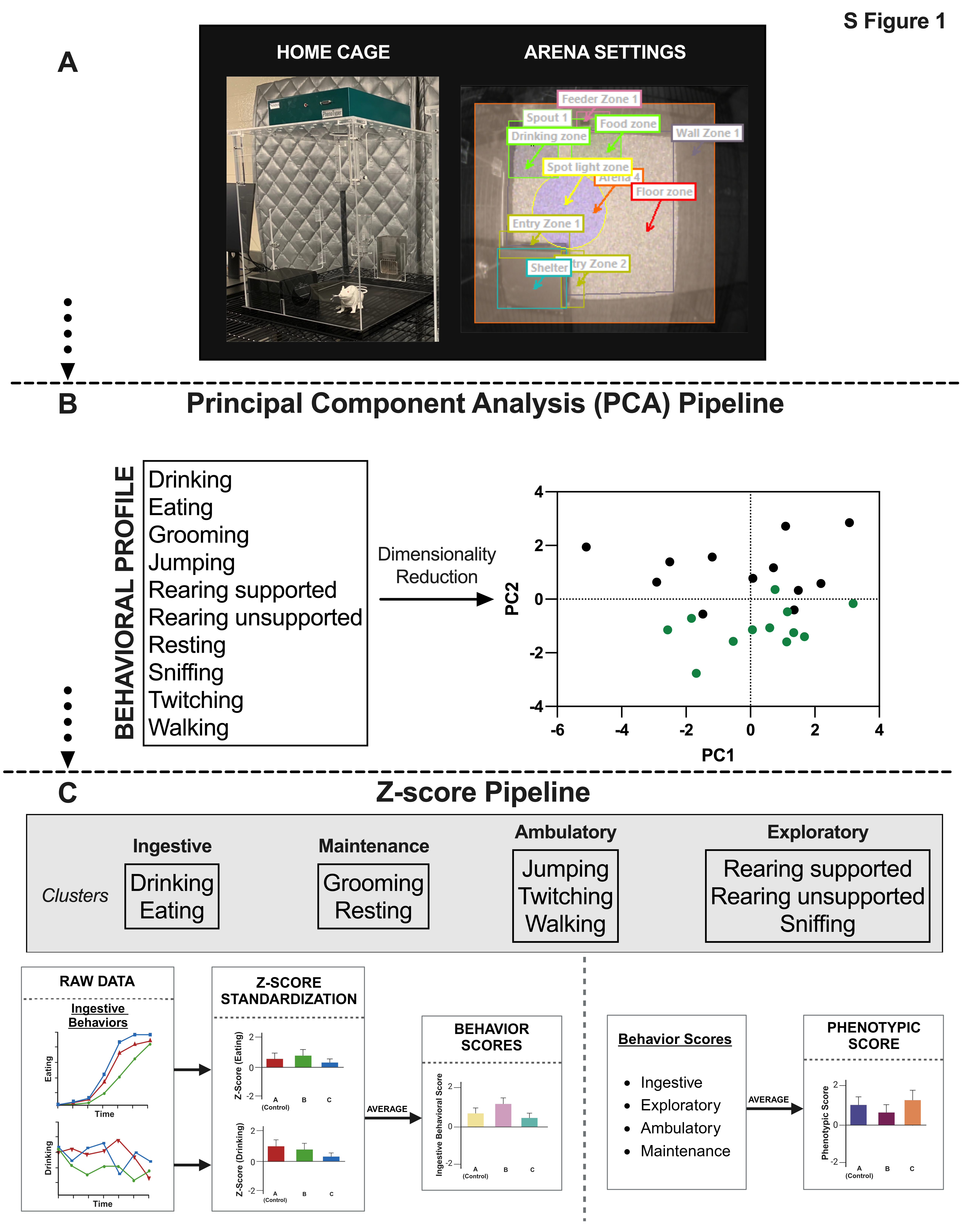

Supplement: Supplementary file 2 — Figure S1 [file BRB3-14-e3482-s004.tiff]

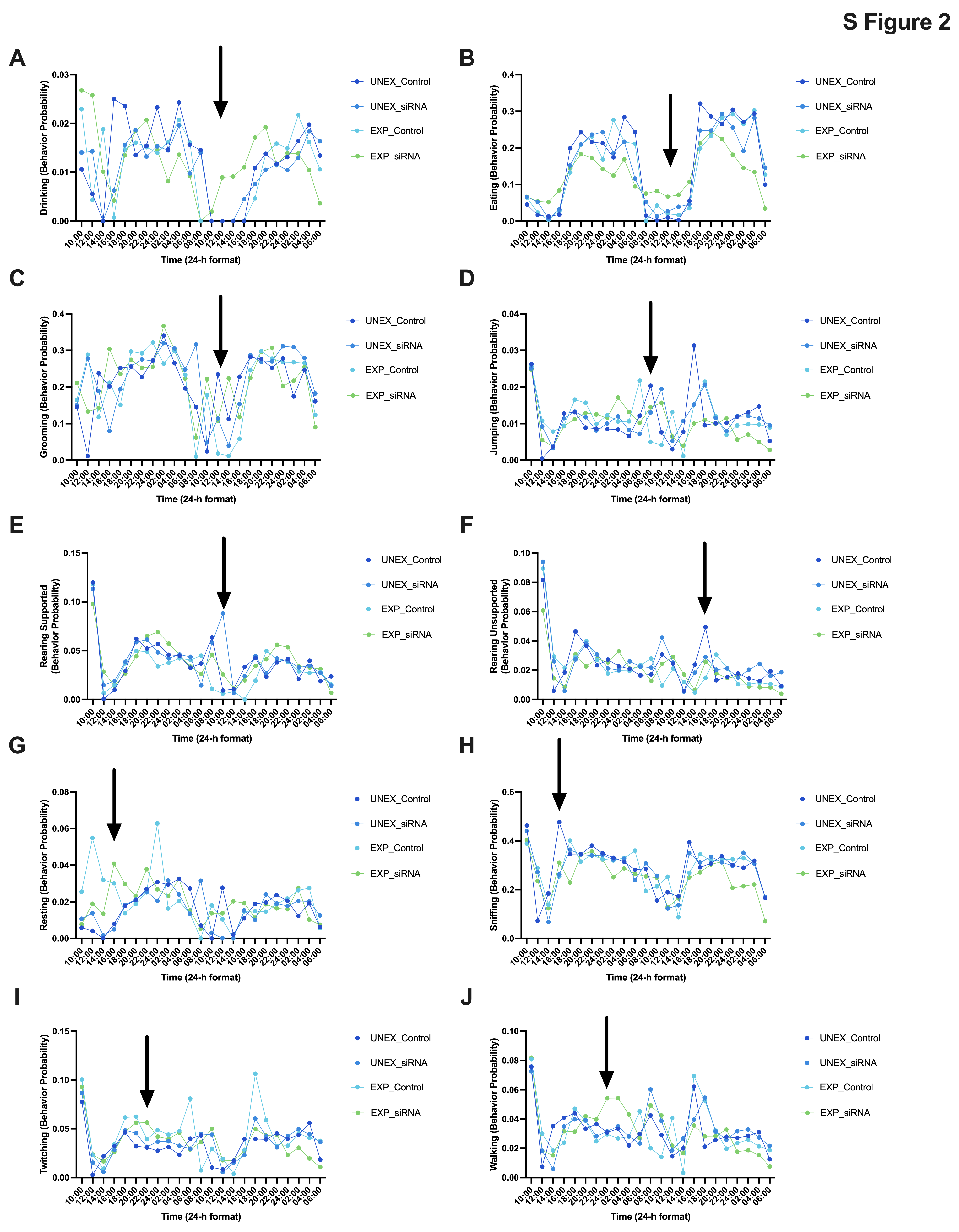

Supplement: Supplementary file 3 — Figure S2 [file BRB3-14-e3482-s007.tiff]

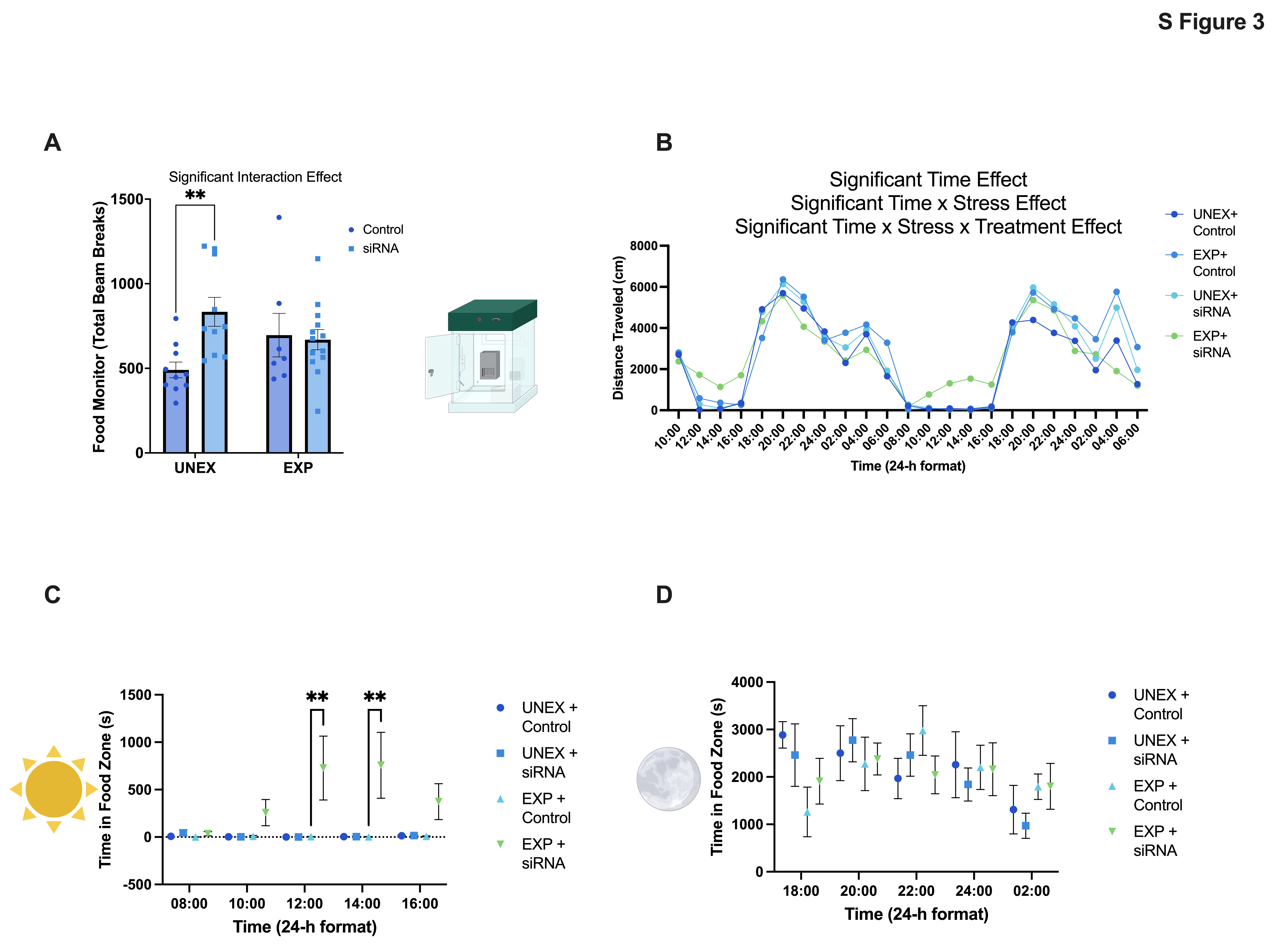

Supplement: Supplementary file 4 — Figure S3 [file BRB3-14-e3482-s008.tiff]

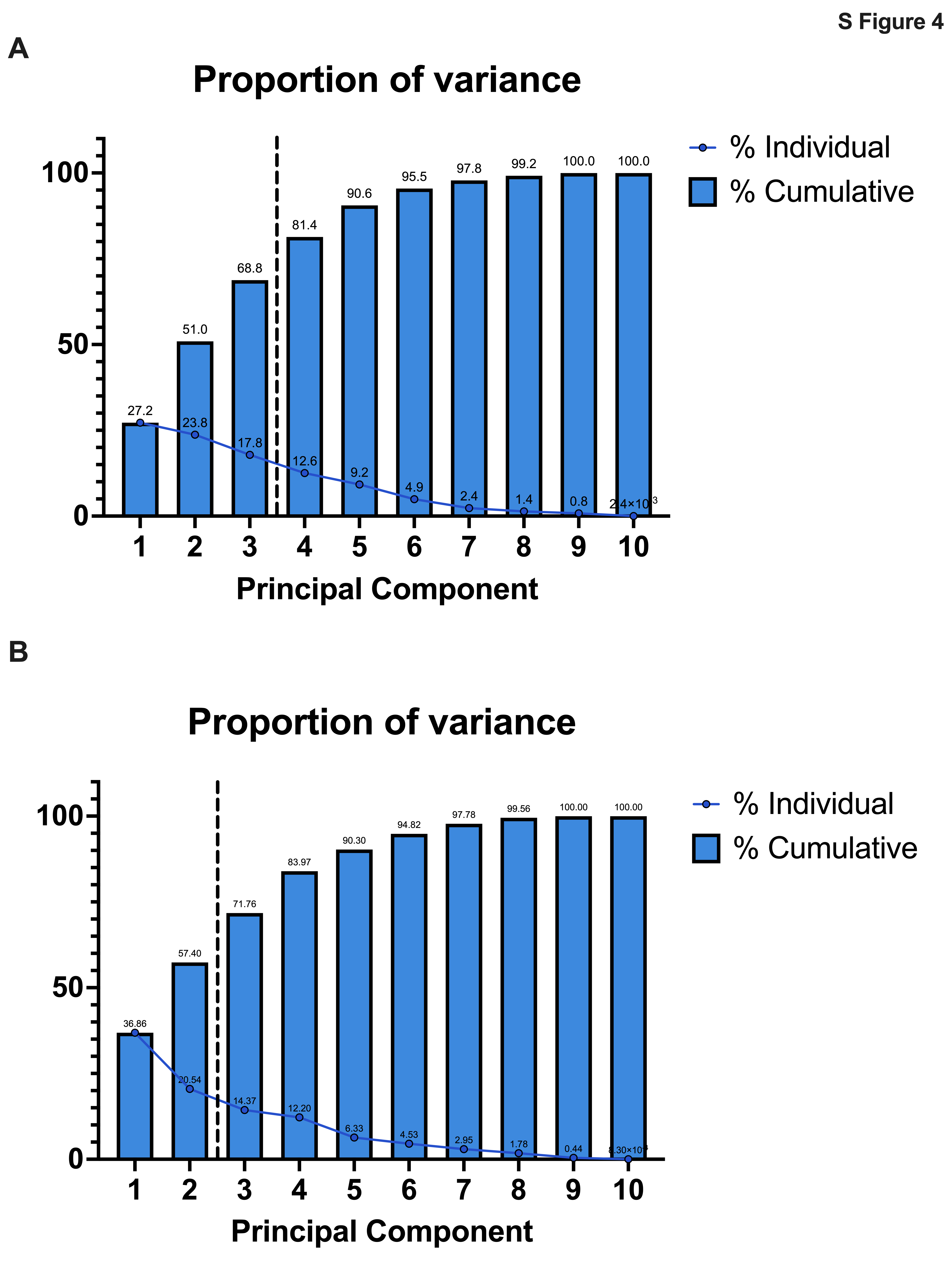

Supplement: Supplementary file 5 — Figure S4 [file BRB3-14-e3482-s003.tiff]

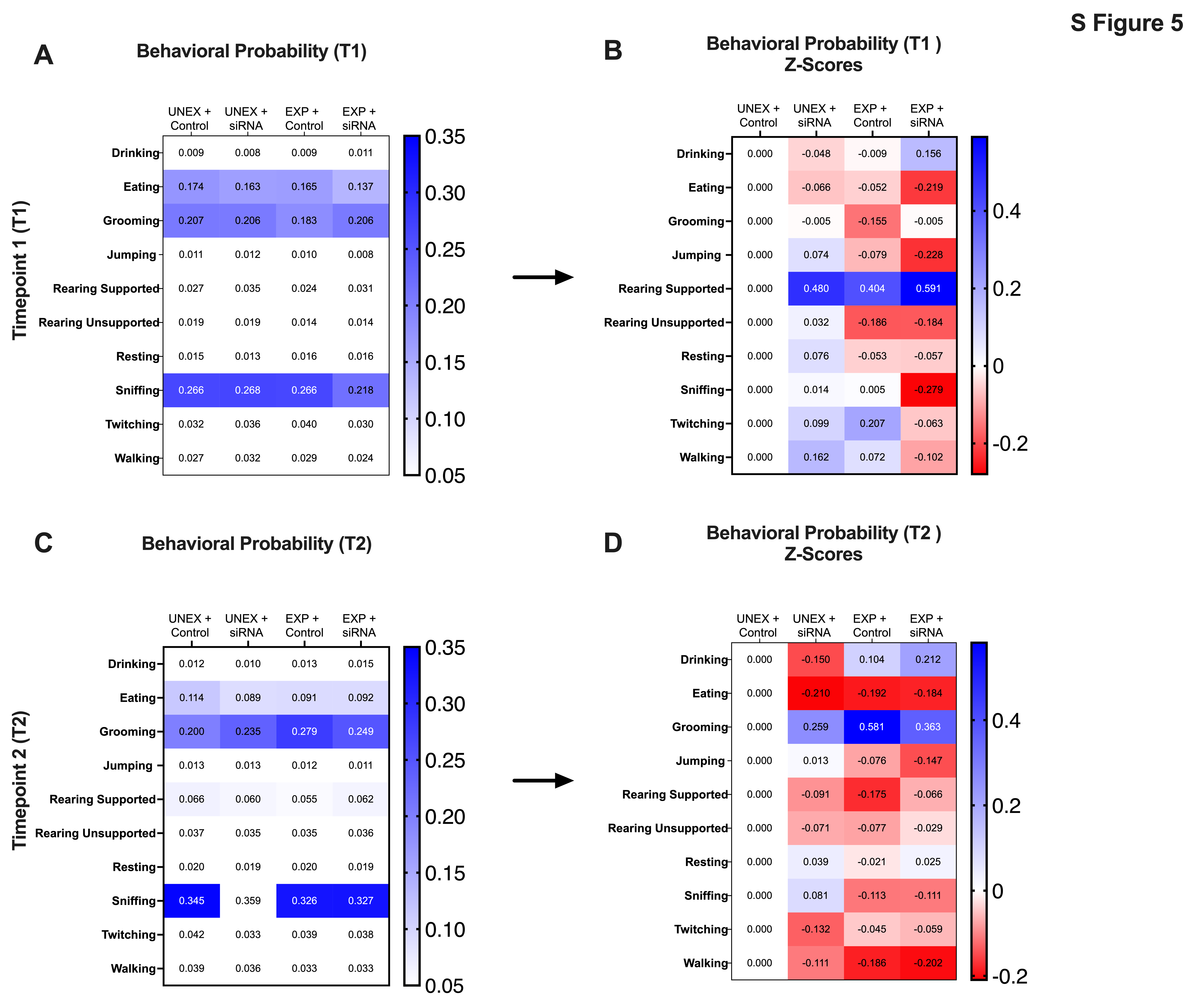

Supplement: Supplementary file 6 — Figure S5 [file BRB3-14-e3482-s005.tiff]

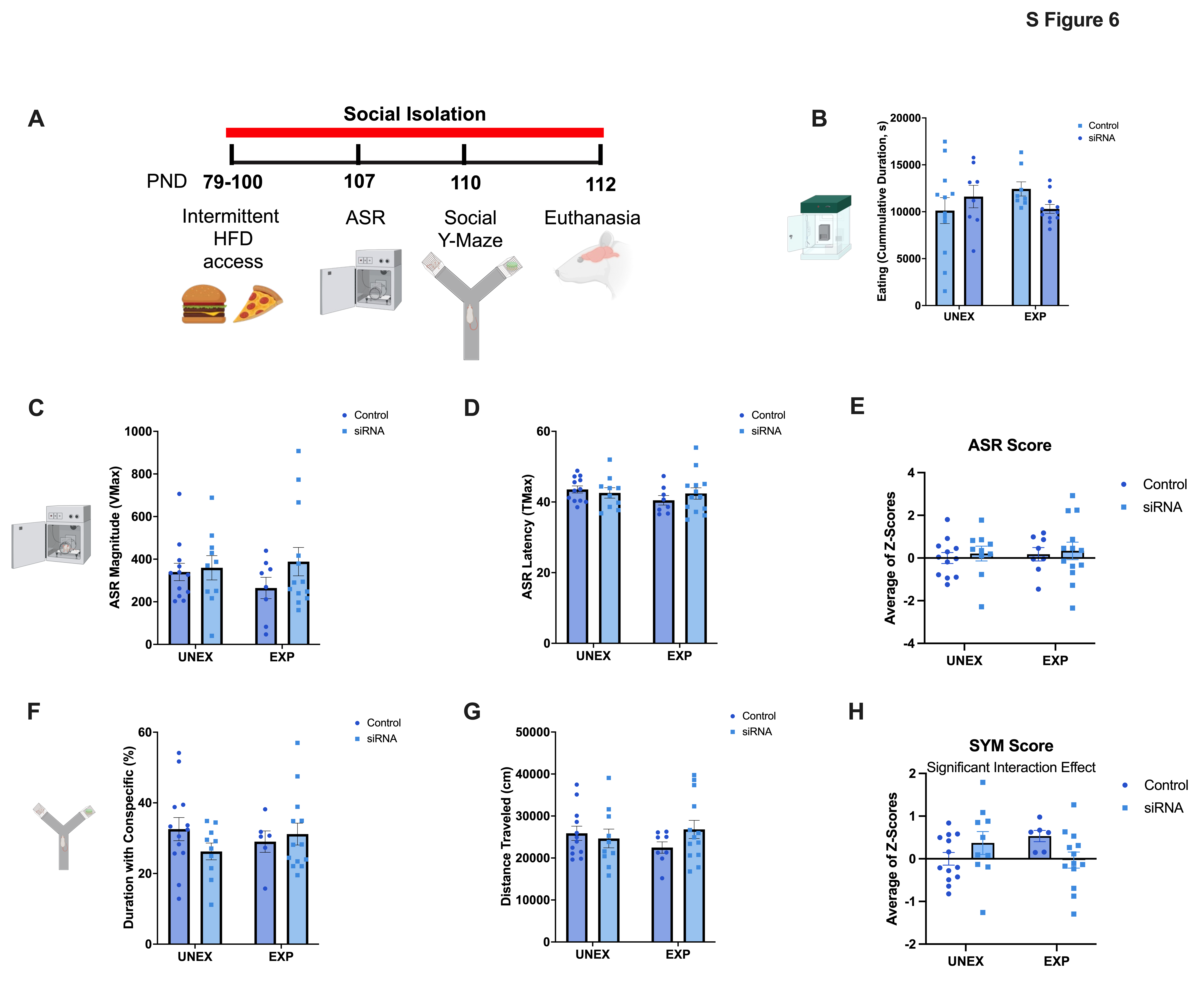

Supplement: Supplementary file 7 — Figure S6 [file BRB3-14-e3482-s001.tiff]

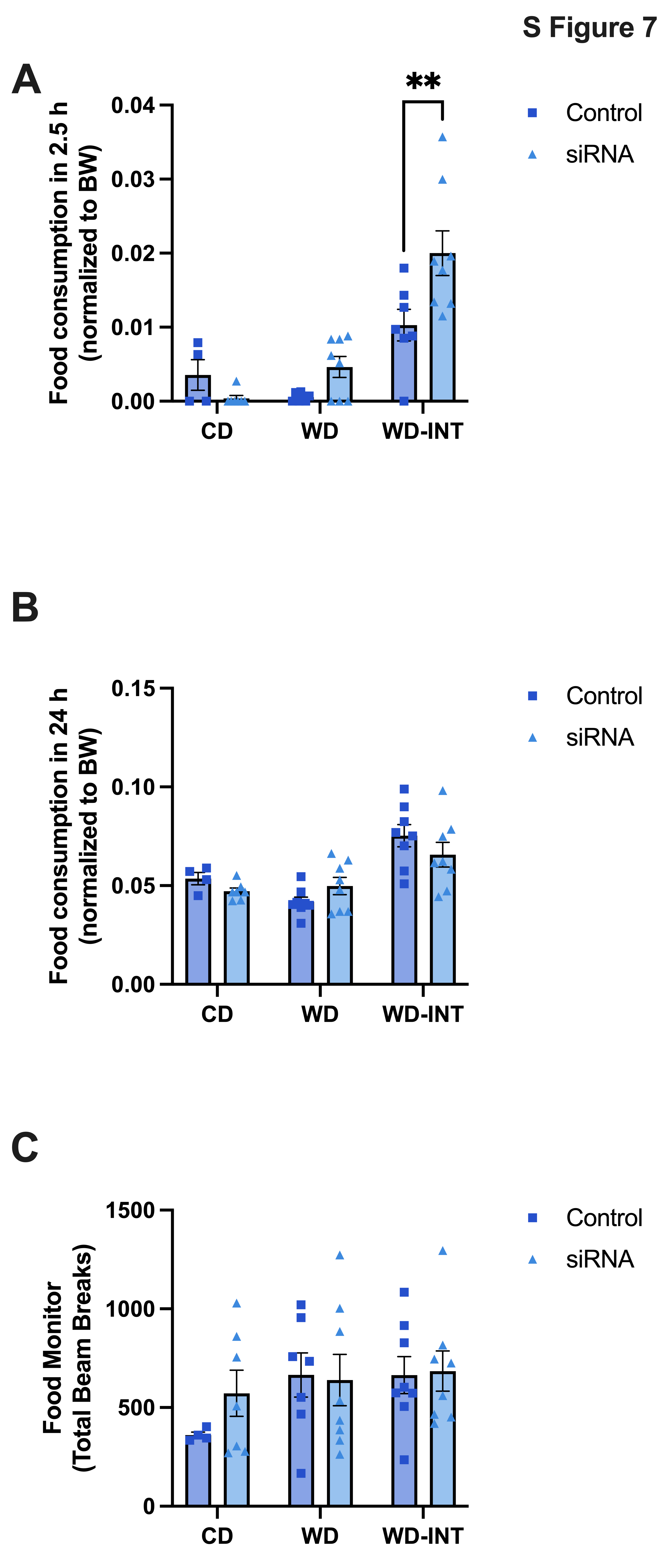

Supplement: Supplementary file 8 — Figure S7 [file BRB3-14-e3482-s009.tiff]

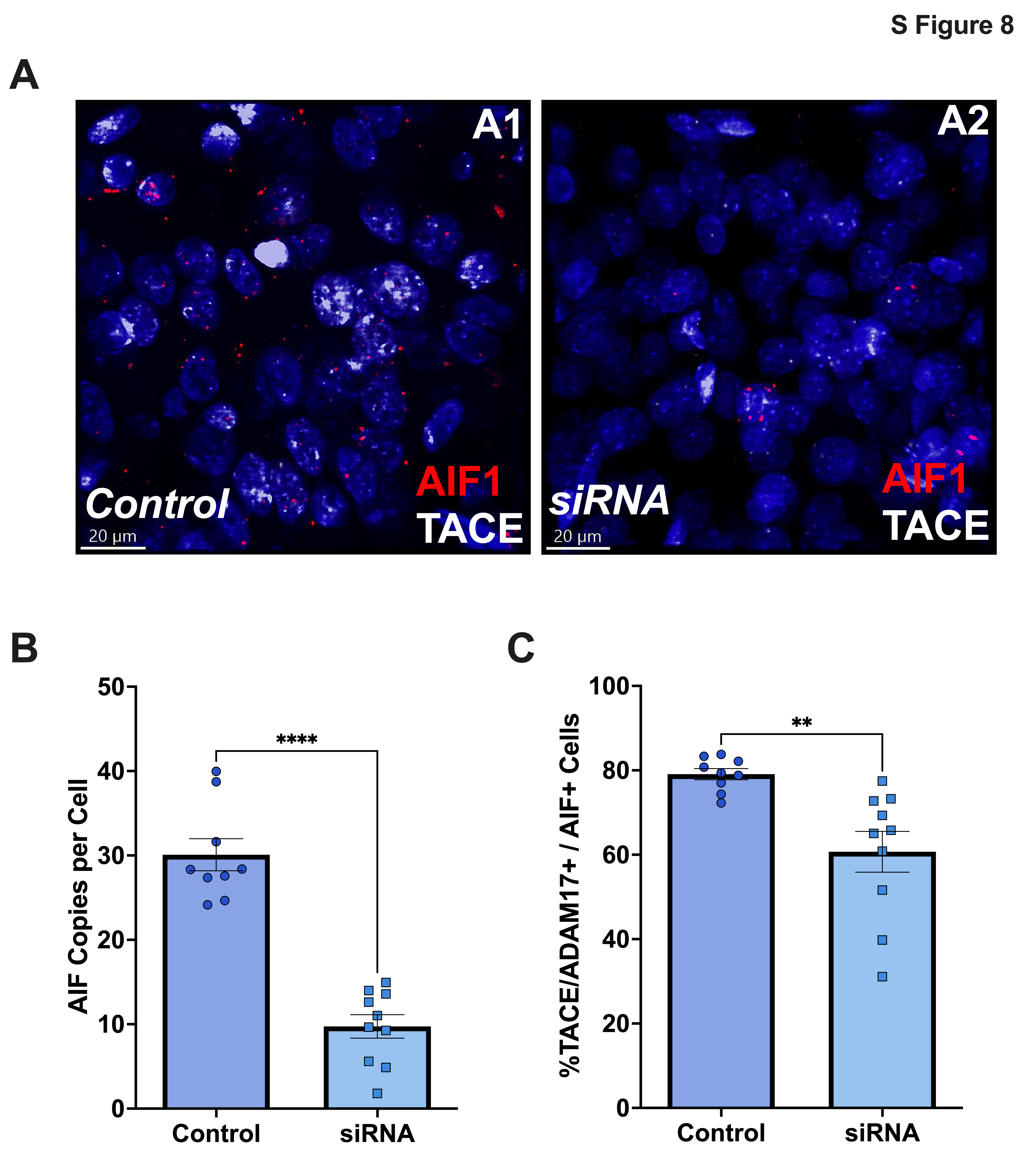

Supplement: Supplementary file 9 — Figure S8 [file BRB3-14-e3482-s002.tiff]
